# A statistical theory of the strength of epidemics: an application to the Italian COVID-19 case

**DOI:** 10.1098/rspa.2020.0394

**Published:** 2020-12-23

**Authors:** Gabriele Pisano, Gianni Royer-Carfagni

**Affiliations:** 1 Construction Technologies Institute - Italian National Research Council (ITC-CNR), Viale Lombardia 49, 20098 San Giuliano Milanese, Milano, Italy; 2 Department of Engineering and Architecture, University of Parma, Parco Area delle Scienze 181/A, 43100 Parma, Italy

**Keywords:** mathematical epidemiology, probabilistic mechanics, Weibull statistics, fracture mechanics, COVID-19

## Abstract

The proposed theory defines a relative index of epidemic lethality that compares any two configurations in different observation periods, preferably one in the acute and the other in a mild epidemic phase. Raw mortality data represent the input, with no need to recognize the cause of death. Data are categorized according to the victims’ age, which must be renormalized because older people have a greater probability of developing a level of physical decay (human damage), favouring critical pathologies and co-morbidities. The probabilistic dependence of human damage on renormalized age is related to a death criterion considering a virus spread by contagion and our capacity to cure the disease. Remarkably, this is reminiscent of the Weibull theory of the strength of brittle structures containing a population of crack-like defects, in the correlation between the statistical distribution of cracks and the risk of fracture at a prescribed stress level. Age-of-death scaling laws are predicted in accordance with data collected in Italian regions and provinces during the first wave of COVID-19, taken as representative examples to validate the theory. For the prevention of spread and the management of the epidemic, the various parameters of the theory shall be informed on other existing epidemiological models.

## 1. Introduction

Mathematical models have been used for almost a hundred years in epidemiology. In their celebrated seminal work Kermack & McKendrick [1] proposed the susceptible–infectious–recovered (SIR) model, detailing how a virus is transmitted from an infected person to a healthy one [2,3]. By introducing the probability that an individual is still infected after a certain time and that a susceptible is contacted by an infected at that stage and becomes infected, integral–differential equations can describe the kinetics of the epidemic, that is, the variation over time in the number *S* of the susceptible to infection, of the infected *I* and of the removed *R*, a compartment that includes the recovered, the immune and the dead as a result of the disease. This rationale has been developed, modified and widely adopted [4–10], also within a probabilistic framework [11], but the basis of the theory remains essentially the original one.

The recent COVID-19 emergency has demonstrated the importance of the SIR model, and its developments, in evaluating the severity of an epidemic through parameters such as the basic reproduction index *R*_0_. Epidemic curves for infected people allow the spread of the epidemic to be followed and the model can evaluate and predict the effects of actions such as social distancing, use of masks and lockdown. However, a major problem consists in determining the exact number of infected people, because not the whole population can be tested and the number of removed is difficult to assess. The various agencies, both public and private, have provided strongly contrasting numbers, at least in Italy. Therefore, any method to evaluate the strength of the epidemic founded on the analysis of epidemic curves, such as the epidemic growth rate [12] or the reproduction number [13], may suffer from uncertainty in the input data. Estimating the growth rate from the epidemic curve can also be objectively difficult, particularly for fast-spreading epidemics for which the estimation may be subject to over-fitting because of the limited number of available data points, which also limits the choice of models for the epidemic curve [14].

Other methods directly analyse mortality data. Sorting out the deaths attributable to the epidemic from those due to other causes is, in principle, simpler than determining the infected of the entire population, given the fewer cases to consider. Despite this, the experience of COVID-19 in Italy has confirmed a difficulty: the *official* number of deaths attributed to the epidemic in its acute phase is about half the increase in deaths with respect to previous years. According to some experts [13,15], the excess mortality rates with respect to pre-epidemic conditions of previous years, regardless of causes of death, should be considered the most reliable (and least assumption laden) records of the effect of the epidemic, rather than the number of infected, or the number of dead specifically from the virus, which are difficult to determine.

Epidemics such as that from COVID-19 are generally associated with disproportionate mortality among the world’s population [16]. It has often been proposed that, as an index of the strength of the epidemic, the differential mortality rate [17] should be considered. This is usually expressed as the ratio between the number of deaths in the *acute* epidemic condition and the number in previous years [18–20]. Here, we aim to demonstrate that the age of the victims also needs to be considered. The paramount role of the demographic structure in the prediction of the expected number of deaths has been confirmed by recent studies [21], as the division by age is more important than the structure of families [22] in the analysis of the social contacts that influence the spread of a virus [23]: social distancing and other policies to slow transmission should always consider the age composition of local and national contexts. We propose a new way to quantify the strength of an epidemic starting from a particular statistical treatment of measurable mortality data in a certain territory, within a given period of observation, which considers the age of the victims.

The fight against epidemics requires a *multi-disciplinary* approach involving integrated cycles of prevention, response and recovery to support epidemiology [24]. Remarkably, the proposed theory has conceptual affinities with consolidated models in the mechanics of materials, to which it may be useful to refer. In 1939 Weibull [25] proposed a theory of the strength of a structure made of a brittle material based on the idea that it contains inherent flaws of random spatial distribution, shape and size. The risk of fracture at a prescribed stress level is therefore determined by the probability that a large-enough defect exists which will propagate catastrophically at that level of stress. The flaw shape is typically assumed to be a flat slit (crack) of size *δ*. If *σ* denotes the stress normal to the crack plane, the *toughness criterion* of linear elastic fracture mechanics (LEFM) dictates that the crack will grow catastrophically (in mode I) when the so-called stress intensity factor *K*_*I*_ = *Yσδ*^1/2^, where *Y* is a constant determined by the geometries of the structure and the crack, reaches the characteristic material’s fracture toughness *K*_*Ic*_ [26]. The Weibull model conceptualizes the structure as a chain composed of equally stressed links, and the resistance of the chain to fracture is limited by the strength of the weakest link. The expected strength is therefore a decreasing function of structural size (size effect [27]), because as the length of the chain increases there is a concomitant increased probability of the presence of a weak link.

The statistical approach and the balance laws underlying this theory of structural strength may also suggest a new statistical theory for the strength of the epidemic, where ‘strength’ now indicates a quantitative measure of lethality with respect to the ‘weakness’ of individuals. Towards this end, it is of paramount importance to define what is the counterpart, for the human body, of the size of the stressed structure (the length of the chain), now associated with the probability of developing a certain level of ‘physical damage’, which induces the potential presence of pathologies and co-morbities critical for the viral infection. We suggest making this correlation, which should be informed by existing epidemiological models, with the age of the individuals. This, however, needs to be renormalized in order to take into account that older people have a higher probability than younger people of having a ‘damaged’ body and, hence, to die as a result of the virus. Such a renormalization, which depends upon genetics, quality of life and efficiency of the health system, can be calibrated starting from mortality data, categorized by age at death, on the basis of calibration criteria naturally derived from the theoretical framework.

The probability of developing a certain level of physical damage specific to the patient is statistically defined as a function of the patient’s renormalized age. A criterion of death is thus proposed, which relates mathematical variables representing the lethal force of the epidemic, its level of spread and our capacity of treating the disease (efficiency of intensive care services, medications, treatments, diagnostic tests, vaccines), as a function of the physical damage of the body. From the theoretical modelling of the probability of death, the strength of the epidemic can be measured. The evaluation can only be *comparative*, in the sense that any two configurations, defined by the respective mortality, are compared in terms of the strength of the epidemic of one state with respect to the other, through an index that, for this reason, is referred to as the *relative index of epidemic*. This index may be calculated from data collected in a certain week or month of a year of acute epidemic, and in the corresponding period of previous years, characterized by milder or less lethal epidemics. The accurate estimate of this index over time and place is useful to follow the spread of the virus, to recognize the emergence of autonomous outbreaks and, even more importantly, to define the timing of appropriate countermeasures such as lockdown (prevention of spread) and to decide when, where and how to lessen the adopted countermeasures (management of the epidemic).

An application is proposed for the Italian COVID-19 case, considered as a good example to validate a theory that is, indeed, very general. Italy was the first country in the Western world in which COVID-19 spread, so much so that the Italian government was first called upon to provide measures for the control of outbreaks, at both the local and national level, in order to avoid the collapse of the health system, which nevertheless occurred in some territories. Furthermore, the various Italian regions differ in age structure, life expectancy and quality of the environment. Therefore, the Italian case represents a laboratory in which to test the proposed theory in various scenarios. The relative index of epidemic has been calculated by comparing weekly and monthly mortality tables during the first wave of COVID-19 (January–June 2020) with corresponding periods of previous years. Considering territories of different sizes (regions and provinces), we estimate the epidemic flow over time, the emergence of autonomous outbreaks and the effect of the imposed lockdown.

The theoretical framework is presented in §2a, referring to §3a for the mathematical calculations. The epidemiology of COVID-19 in Italy provides the scenario discussed in §2b, for which the details on the analysed mortality data are indicated in §3b. The potential of the proposed approach is discussed in §4, by comparing its results with those obtainable by analysing the simple differential mortality. Through the parameters of the model it is possible to evaluate how the epidemic has spread and to predict its development as a consequence of control actions and improved treatments. However, the way in which the parameters of the theory should be related to the many variables that detail the kinetics of the epidemic is currently an open issue.

## 2. Results

### (a) The theoretical framework

We start from the assumption that the life of a person is represented by a chain composed of *life-segments* that represent the rings in the chain analogy. The number of rings is successively increased to reflect the ageing process, as rings represent the *monads* associated with human degradation. One may consider that a life-segment is a solar year of age, or submultiple, but this view is too simplistic. ‘It is mathematically demonstrable that the concept of time is closely related to age: time passes faster for old people’. This quote, by the American writer Alvin Toffler (1928–2016), introduces the idea that the usual lifetime unit, e.g. the solar year, cannot indicate the reference scale to measure the ageing process throughout the whole of human life. If a life-segment represents the nominal unit associated with *one* spot where potential damage may develop with *equal probability*, the number of life-segments contained in one solar year of age should be higher for an older individual than for a younger one.

Let Δ*A*_*n*_ denote the reference *nominal* life-segment. The *real age* *A*_*r*_, expressed as the number of solar (real) time-segments Δ*A*_*r*_, is rescaled to the *nominal age* *A*_*n*_, equal to the number of Δ*A*_*n*_, through a *renormalization* group, expressed by *A*_*n*_ = *F*(*A*_*r*_). Elaborating the data on mortality recorded in Italy, we are led to consider a function *F*(*A*_*r*_) of the form

2.1 $$A_{n}=F(A_{r})=A_{r}+{\left\langle A_{r}-45\right\rangle }_{+}^{\gamma_{1}}+{\left\langle A_{r}-70\right\rangle }_{+}^{\gamma_{2}},\;\gamma_{1},\gamma_{2}>0,$$

where *A*_*r*_ and *A*_*n*_ are expressed in the number of solar and nominal years, respectively, and 〈 · 〉_+_ denotes the positive part function.^1^ Hence, there are two step-changes in life, the first at 45 and the second at 70 solar years of age, beyond which, roughly speaking, ‘each year counts more’.

The form of the renormalization law (2.1) has been derived from direct calibration. While postponing the detailed explanation to the end of this section, we anticipate that the proposed theory predicts a precise distribution of mortality rates according to the nominal age of the individuals, which rescales according to the force of the epidemic and its degree of spread. Analysing the mortality tables, the renormalization law has been calibrated by requiring its compliance with the distribution predicted by the theory; the observed rescaling consequent to the epidemic provides a confirmation of its soundness. The function (2.1) was found within the class of functions that prescribe an exponential step-change at two chronological ages, whose parameters have been determined by the best fit with the data. In general, the renormalization *A*_*n*_ = *F*(*A*_*r*_) may depend on, among other factors, race, genetic heritage, environmental factors, quality of life and gender (male or female). For example, analysing the data from Italy it has been evidenced that, for the same chronological age, the nominal age is lower in the territories on the sea than in the industrial hinterland. Although a generalization is possible, the renormalization laws that will be considered in the following have been evaluated from the analysis of mortality data within each analysed Italian region, considered as a *homogeneous ensemble*—a subset of the national territory.

With advancing age, deterioration of the organism increases and therefore the probability of developing critical pathologies increases as well. Body decay due to ageing, which may be referred to as *human damage* by analogy with the term in mechanics, is defined by a state variable and depends, in probabilistic terms, on the nominal age of the individual. The probability of death therefore derives from the probability of developing a level of human damage that is critical to the severity of the epidemic, according to a yield criterion. Referring to §3a for the detailed calculations, here the basic results of the theory are summarized and discussed.

Suppose that the level of human damage is measured by one variable *δ*. The value of *δ* shall increase with age, and the term of comparison is defined by the number of nominal life-segments. The higher the number, the higher the probability of developing critical pathologies that may lead to death. However, there are differences among individuals, so that the dependence of *δ* upon (nominal) age needs to be considered in statistical terms. Given a sample consisting of a number of individuals, each one experiences a different level of damage, so the percentage of individuals that have the same level of damage defines the probability of finding that level of damage in one individual.

A probability function can be defined with reference to the single life-segment, so that the effect of ageing derives from increasing the number of segments. We postulate the existence of a statistical law *à la* Pareto, as per (3.1) in §3a, expressed by the probability density function *p*_Δ*A*_(*δ*) = *Cδ*^−*α*^, where *C* > 0 and *α* > 1 are constants, of developing the level of damage *δ* with reference to *one* segment of nominal life. There is certainly a minimum level *δ*_min_ of physiological damage, otherwise it would be theoretically possible to live forever. Therefore, the normalization considered in detail in §3a yields, *C* = (*α* − 1)/(*δ*_min_)^−*α*+1^.

Originally applied to describing the distribution of wealth in a society, Pareto statistics are commonly applied in the description of social, scientific, geophysical, actuarial and many other observable phenomena. Reasoning as in §3a, one obtains that the probability of finding in Δ*A*_*n*_ a level of damage greater than or equal to *δ* decreases with *δ* and reads $P_{\Delta A_{n}}^{\geq }(\delta )={\left(\delta_{\text{min}}/\delta \right)}^{\alpha -1}$ for *δ* ≥ *δ*_min_. In more general terms, the function $P_{\Delta A_{n}}^{\geq }(\delta )$ shall be monotonically decreasing, equal to 1 at *δ* = *δ*_min_ and tending to zero as *δ* → +∞. Also, it shall be invariant for scale transformations, i.e. it is a function of *δ*/*δ*_min_. The assumed power-law dependence is the simplest that can be considered and intrinsically enjoys the property of self-similarity, which has wide applications in the description of physical phenomena [28]. More complicated statistical distributions could be considered from the mathematical modelling of human physiology, but the purpose here is to define the theory in the simplest case.

Likewise, increasing the number of life-segments increases the probability of damage. The statistical calculations in §3a provide the probability of developing *δ* in the whole nominal life *A*_*n*_, composed of *A*_*n*_/Δ*A*_*n*_ lifetime segments, in the form

2.2 $$P_{A_{n}}^{\geq }(\delta )=1-\exp\left[-\frac{A_{n}}{\Delta A_{n}}{\left(\frac{\delta }{\delta_{\text{min}}}\right)}^{1-\alpha }\right].$$

This expression is dimensionally consistent.

We further postulate that the ‘force’ of the epidemic is measured by another variable *σ*, which accounts for the fact that some form of epidemics may be more lethal than others. In the simplest case, we assume the LEFM-like [26] *death criterion*

2.3 $$Y\sigma \;{\left(\frac{\delta }{\delta_{\text{min}}}\right)}^{1/\beta }-K_{Ic}=0\Rightarrow \frac{\delta }{\delta_{\text{min}}}={\left(\frac{K_{Ic}}{Y\sigma }\right)}^{\beta }.$$

This is a balance law where the level of the epidemic, measured by the product *Yσ*, where *Y* is a coefficient, is multiplied by the number ${\left(\delta /\delta_{\text{min}}\right)}^{\beta }$ and compared with a limit value *K*_*Ic*_, carrying the same dimensions of *Yσ*, which represents the ‘human toughness’, i.e. the ability of the human body to react to infections.

The variable *Y* measures the ‘level of spread of the epidemic’, since the risk of death is not only associated with the force of the epidemic but also with the possibility of being infected. With reference to the SIR model [1], *Y* could be associated with the ratio between the number of infected *I* and the number of susceptible *S*. Consequently, it is a function of time and depends upon the actions [29] that could be taken to control the epidemic (social distancing, reduced mobility, use of masks, lockdown). The variation of *Y* in time could be assessed through the equations of the SIR model describing the kinetics of the epidemic spread, taking into account the possible interventions [12].

The quantity *Yσ* is multiplied by the number (*δ*/*δ*_min_)^1/*β*^, which considers the effects of human damage. Observe that, since *δ*/*δ*_min_ ≥ 1, by increasing *β* the effect of human damage becomes milder. This means that *β* synthetically takes into account our capacity to cure the disease, in terms of availability of beds in intensive care services, medications, treatments, oxygen, diagnostic tests, etc. How all these factors affect the value of *β* can be decided by evaluating their effects on mortality rates for patients who have the same level of human damage *δ*/*δ*_min_, associated with factors specific to the patient (e.g. co-morbidity). Again, the power law is the simplest dependence that can be assumed while respecting dimensional invariance, but determining the expression of *β* as a function of the capacity of the health system certainly requires a sophisticated analysis of available data, which is not done here.

Determining the specific expression for the various variables in the death criterion (2.3) requires a deep understanding of the biological mechanisms that cause severe infections, which for COVID-19 certainly involve a cytokine storm, enhancement of antibody dependence, immune history and T-cell immunity. However, the presented theory is general and not limited to a particular type of epidemic. Our interest here is to evaluate the consequences of this theory from mathematical deductions, without entering into specific details that go beyond the competence of the authors. From a mathematical point of view, it is important to observe that *Yσ*/*K*_*Ic*_ ≤ 1 since *δ*/*δ*_min_ > 1. In particular, the situation in which *Yσ*/*K*_*Ic*_ = 1 is the limit situation in which the physiological level of damage *δ*_min_ is sufficient to cause death. Under this condition, everybody dies from the epidemic! Hence, under ordinary conditions it is expected that *Yσ*/*K*_*Ic*_ ≪ 1.

Substituting in (2.2), the *probability of death* as a function of *σ* and the real age *A*_*r*_ is

2.4 $$P^{D}(A_{r},\sigma )=1-\exp\left[-\frac{F(A_{r})}{\Delta A_{n}}{\left(\frac{Y\sigma }{K_{Ic}}\right)}^{m}\right],$$

which is again dimensionally consistent. This is a two-parameter Weibull distribution with shape-parameter *m* = (*α* − 1)*β* and scale-parameter *K*_*Ic*_/*Y*. The nominal age *A*_*n*_ = *F*(*A*_*r*_) determines the ‘size effect’ consequent to the fact that the higher the number of life-segments of the individual, the higher the risk of death.

In the mechanics of brittle materials [30], the counterparts of *δ* and *σ* are the size of the existing cracks and the stress level, respectively, whereas *Y* is the shape factor for the crack [31,32]. The death criterion recalls the criterion for catastrophic crack propagation in LEFM [26], for which *β* = 2 and *K*_*Ic*_ is the material fracture toughness. The nominal life represents the size of the material body, which affects the probability of failure since the higher it is, the higher the probability of finding a crack of critical size with respect to the applied stress, according to classical LEFM.

In order to quantify the severity of the epidemic state, note that (2.4) may be re-written as

2.5 $$\ln\ln\left[\frac{1}{1-P^{D}(A_{r},\sigma )}\right]=\ln\left[\frac{F(A_{r})}{\Delta A_{n}}\right]+(\alpha -1)\beta \ln\left[\frac{Y\sigma }{K_{Ic}}\right],$$

which represents a straight line *Z* = *X* + *Q* in the plane *Z* = lnln{[1 − *P*^*D*^(*A*_*r*_, *σ*)]^−1^} − *X* = ln[*F*(*A*_*r*_)/Δ*A*_*n*_]. This plane is somewhat the counterpart, in this theory, to the Weibull plane [25], which is commonly used to interpret the strength of brittle material on a statistical basis and, because of this, will be referred to as the *epidemic Weibull plane*.

It is remarkable that an immediate verification of the validity of the proposed theory consists in verifying that the measured mortality rates, ordered by nominal age, are actually aligned, with unit slope, in the epidemic Weibull plane, regardless of the parameters *α*, *β*, *Y*, *σ*, *K*_*Ic*_. Conversely, the fact that the experimental points must be aligned in the Weibull plane provides the criterion for determining the normalization law *A*_*n*_ = *F*[*A*_*r*_]. The procedure consists in finding the renormalization law that provides the best fit to the experimental points in one particular state of the epidemic and, then, verifying that the same normalization provides a reasonable alignment in other states as well. It will be shown in the following sections that the renormalization (2.1), determined by analysing data before 2020, satisfies this condition for the analysed regions and provinces in Italy in various stages of the evolution of the COVID-19 epidemic. The same normalization holds in the 2020 conditions of acute epidemics: this confirms the theory.

The height of the interpolation line in the epidemic Weibull plane, which is measured by the value of its intercept *Q* with the ordinate axis, provides a measure of the distributions of the deaths by renormalized age, as a consequence of the epidemic. The higher the line, the higher the death rate among older people compared with younger people, and vice versa. Indeed, the strength of the proposed model is that it is not limited to taking into account the raw number of deaths, but *measures the shift in mortality rates between categories defined by the nominal age of the dead*. In fact, the probability function (2.2) assigns to older people a higher probability of developing critical pathologies as a result of physiological damage; on the other hand, the death criterion (2.3) provides a close correlation between the level of human damage, measured by the quantity *δ*/*δ*_min_, and the force of the epidemic *σ* and its diffusion level *Y*. Because of this, older people are more likely to die of infection than younger people; hence, the analysis of age-sorted death rates can indicate the severity level of the epidemic. In other words, this theory detects any increase in deaths in older people compared with younger people, rather than a uniform increase in deaths that leaves the relative rates unchanged in the various age groups, and associates this specific event with the effects of an epidemic.

The height of the line (2.5) in the epidemic Weibull plane is defined by the quantity

2.6 $$Q=(\alpha -1)\beta \ln\left[\frac{Y\sigma }{K_{Ic}}\right],$$

where, we recall, *α* > 1, *β* > 0, *Yσ*/*K*_*Ic*_ < 1. Hence, in general *Q* < 0. The parameter *α* that affects the renormalization of human age, as well as *K*_*Ic*_, which is a measure of human toughness, is in general independent of the level of epidemics. As the values of *σ* and/or *Y* increase, so that the ratio *Yσ*/*K*_*Ic*_ approaches the unit value, *Q* tends to become null, i.e. the intercept with the ordinate axis of the interpolation line approaches the origin from below. Likewise, an increase in *β*, which measures our capability of treating the disease, provides the lowering of this line. The condition *Q* = 0 is associated with the extreme limit where *Yσ*/*K*_*Ic*_ = 1, where the whole population dies from the epidemic because, from (2.3), the minimum level of damage *δ* = *δ*_min_ is sufficient to cause death.

For the above, an effective measure of the level of epidemics could be obtained by measuring the inverse of the absolute value of *Q*, i.e. −1/*Q*. However, the various parameters that characterize one configuration must be compared with those corresponding to another configuration in order to obtain a quantitative estimate, which can only be obtained in relative terms. Let *β*_0_, *Y*_0_, *σ*_0_ represent the parameters of a configuration, chosen as the reference state, labelled as ‘0’, and indicate with *β*_1_, *Y*_1_, *σ*_1_ the parameters for another state, say ‘1’, which shall be compared with ‘0’. Recall that *α* and *K*_*Ic*_ are generally the same for both configurations, since they depend on the population and are not affected by the epidemic level. From the analysis of the mortality data, it is possible to determine the best-fit line in the epidemic Weibull plane for configurations ‘0’ and ‘1’ and, correspondingly, the intercept on the ordinate axis identified by *Q*_0_ and *Q*_1_, respectively. A relative measure of the strength of the epidemics is thus provided by the *epidemic ratio* *r*, defined as

2.7 $$r=\frac{-1/Q_{1}}{-1/Q_{0}}=\frac{Q_{0}}{Q_{1}}=\frac{\beta_{0}}{\beta_{1}}\;\frac{\ln[\left(Y_{0}\sigma_{0}\right)/K_{Ic}]}{\ln[\left(Y_{1}\sigma_{1}\right)/K_{Ic}]}=\frac{\beta_{0}}{\beta_{1}}\;\frac{\ln[K_{Ic}/\left(Y_{0}\sigma_{0}\right)]}{\ln[K_{Ic}/\left(Y_{1}\sigma_{1}\right)]}.$$

It is clear that when *r* > 1 the level of epidemic in configuration ‘1’ is higher than in configuration ‘0’, because the interpolation line for ‘0’ is higher than for ‘1’. For *β*_0_ = *β*_1_, this condition is met when *Y*_1_*σ*_1_ > *Y*_0_*σ*_0_. On the other hand, for *Y*_1_*σ*_1_ = *Y*_0_*σ*_0_, one has that *r* < 1 (*r* > 1) when *β*_1_ > *β*_0_ (*β*_1_ < *β*_0_): the capacity of curing diseases can greatly alter the effects of the epidemic.

The value of the ratio of epidemics *r* is in general a number close to 1. From a practical point of view, what is important is to evaluate the difference of *r* from the unit and this can be done with the *relative index of epidemic* *I*_*e*_, defined as

2.8 $$I_{e}=100(r-1)=100\frac{Q_{0}-Q_{1}}{Q_{1}},$$

where the factor 100 has been introduced to render the numbers easier to read. According to this rationale, *I*_*e*_ > 0 (*I*_*e*_ < 0) indicates that the ‘strength’ of the epidemic is higher (lower) in situation ‘1’ than in situation ‘0’.

In order to illustrate the potential of the theory, statistically representative samples of the Italian population under conditions of contagion from COVID-19 (configurations ‘1’) will be compared with an ‘ordinary’ condition of comparison (configuration ‘0’). For convenience, we will refer to ‘1’ and ‘0’ as the ‘epidemic’ and ‘pre-epidemic’ configurations, respectively, with the aim of emphasizing that a stronger source of infection, in particular COVID-19, characterizes configurations ‘1’ with respect to ‘0’. It should be remarked, however, that the ‘0’ state is by no means free from infections, although they should be driven by milder forms of epidemic, such as seasonal influenza. The evaluation of the level of epidemic must necessarily be comparative, since the variables *β*, *Y* and *σ* represent dimensional quantities, the value of which can only be determined with respect to suitable units of measurement. Thus, the ratios *Y*_1_/*Y*_0_, *σ*_1_/*σ*_0_, *β*_1_/*β*_0_ denote the value of the variables in configuration ‘1’ when the value in configuration ‘0’ is chosen as the unit of measure. Of course, the choice of the configuration of comparison ‘0’ is quite arbitrary. In the following, various types of configurations ‘1’, corresponding to critical weeks or months in the period January–June 2020, will be compared with the ‘0’ configurations for the same weeks or months of the previous years, prior to the spread of COVID-19.

### (b) Application to the COVID-19 epidemic in Italy

In Italy, two Chinese tourists were recorded as being positive for COVID-19 on 30 January 2020, while the contagion officially started on 21–22 February. The Italian government imposed a national quarantine on 9 March, when Italy had 9172 confirmed cases; however, 10 municipalities in the province of Lodi and one in Padova had already been quarantined since 24 February as a preventive measure. Italians were forced to stay home: movements were only allowed for ‘urgent, verifiable work situations and emergencies or health reasons’; schools and universities were closed; civil and religious ceremonies and any other event were suspended; public and private companies were encouraged to put their employees on leave; restaurants and bars were closed and people had to maintain at least 1 m of interpersonal distance and wear masks. The gradual release of the lockdown started on 4 May, with the reopening of manufacturing activities, bars, restaurants, barbers, beauty parlours and shops on 18 May. Travel between Italian regions and to and from a few foreign countries was gradually allowed from 3 June.

According to the Italian Ministry of Health, deaths increased from 29 on 29 February to 34 767 at the end of June. Using published data, we start by analysing 16 Italian regions, which are considered as homogeneous boxes. Northern Italy was reputed to be the most infected zone; in southern Italy the infection was mild; central Italy was in between. The remaining regions (Basilicata, Molise, Calabria, Sardegna) are not analysed here because the mortality from COVID-19 has been almost zero in these regions, at least so far. The *period of observation* was chosen to be the six-month period corresponding to the first wave of COVID-19: the results are obtained by processing the data for the deaths that occurred in a time interval, which varies according to the type of analysis, that is included in the period January–June 2020 and in an analogous interval in previous years.

We have calculated the *relative index of epidemic* *I*_*e*_, defined in (2.8), on a regional basis. Setting a single month as the period of observation, from January to June, the renormalization *A*_*n*_ = *F*(*A*_*r*_) in pre-COVID-19 conditions, which represents the configuration of comparison (configuration ‘0’), is calibrated from the statistical analysis of the number of dead, sorted by age, in the considered month during the years 2015 to 2019, and calculating the average. These data are published online by the Italian National Institute of Statistics (ISTAT) at https://www.istat.it/it/archivio for many Italian municipalities, which have been grouped by region of origin. The total population at the beginning of March is available online at http://demo.istat.it, together with the demographic structure for each solar year. The population and number of dead were categorized by age by considering nine sets for $A_{r}=\{0\text{--}10,10\text{--}20,\ldots ,80\text{--}90,>90\}$ solar years of age. Within the *i*th set, the probability of death is $n_{i}^{D}$, i.e. the ratio between the number of dead and the peer population. Then, the average probability of death in the period 2015–2019 is calculated for each *i*th set. To pass from a histogram to points on a graph, we consider the age at the centre of each interval (5, 15, …, 85) and 100 for ages greater than 90 solar years. The distribution of (2.4) provides the theoretical scaling between the probabilities of death at any two real ages *A*_*r*,1_ and *A*_*r*,2_, to be compared with measured data. In all the considered regions, the assumed form of renormalization of (2.1) proved accurate. Equating the measured and expected probabilities at one point *A*_*r*,2_, chosen to be *A*_*r*,2_ = 45 because, here, *A*_*r*,2_ = *A*_*n*,2_, we calibrate *γ*_1_ and *γ*_2_ of (2.1). The results of the calibration are detailed in §3b, with specific reference to the region of Lombardia.

Passing to the periods of observation in 2020, which represent configuration ‘1’, at https://www.istat.it/it/archivio one finds the number of deaths sorted by age. Since only the population, but not the demographic structure, is recorded at http://demo.istat.it, we assume that this is equal to that for the year 2019. The theoretical model predicts that the parameters *γ*_1_ and *γ*_2_ do not vary, since the effects of the epidemic are gathered in the product *Yσ* of (2.4). The fitting with the experimental points confirms this finding, with very good approximation. This represents an experimental validation of the theory.

**Figure 1.**
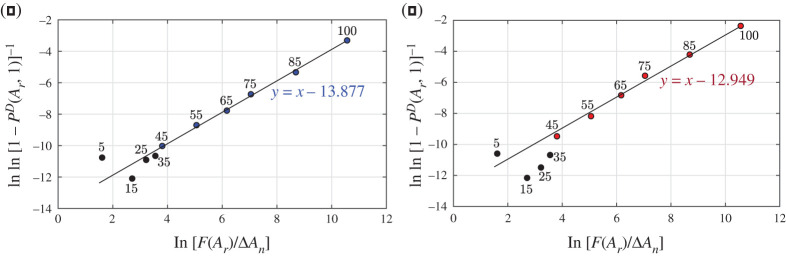
Region of Lombardia. Graphical regressions in the epidemic Weibull plane *Y* = lnln{[1 − *P*^*D*^(*A*_*r*_, *σ*)]^−1^} − *X* = ln[*F*(*A*_*r*_)/Δ*A*_*n*_], with $\Delta A_{n}=1\;\text{year}$, of the probability of death *P*^*D*^(*A*_*r*_, *σ*) as a function of the renormalized age *A*_*n*_ = *F*(*A*_*r*_). Comparison of the measured points (dots) and their linear interpolation in the epidemic Weibull plane according to the theory. (*a*) Conditions before COVID-19 in March (average of years 2015–2019); (*b*) epidemic period of March 2020. The numbers above the dots indicate the solar years of age they refer to. Points corresponding to 5, 15, 25, 35 solar years of age are strongly sensitive to small variations in the number of deaths, which may derive from non-epidemic causes not considered by the theory. (Online version in colour.)

In order to calculate the index *I*_*e*_ we use the graphical construction in the *epidemic Weibull plane*. Figure 1*a*,*b* shows the measured points and the best-fit line in Lombardia in March 2015–2019 (configuration ‘0’) and March 2020 (configuration ‘1’), respectively. The comparison demonstrates that the model excellently predicts the deaths in the middle and older age groups. The poor approximation on the left-hand-side tail can be attributed to the fact that the number of dead is quite limited for younger people, so that even a small variation can provide a noteworthy deviation from the linear trend. It should be recalled that deaths at a young age often occur from non-epidemic causes, such as road accidents, drugs and homicides, which cannot be covered by our theory. The fact that the datum for the 0–9 age group is higher than that for the 10–19 and 20–29 age groups is presumably due to deaths in the first years of life, not associated with the natural degradation of the human body (human damage) and the consequent potential ability to develop a pathology.

**Figure 2.**
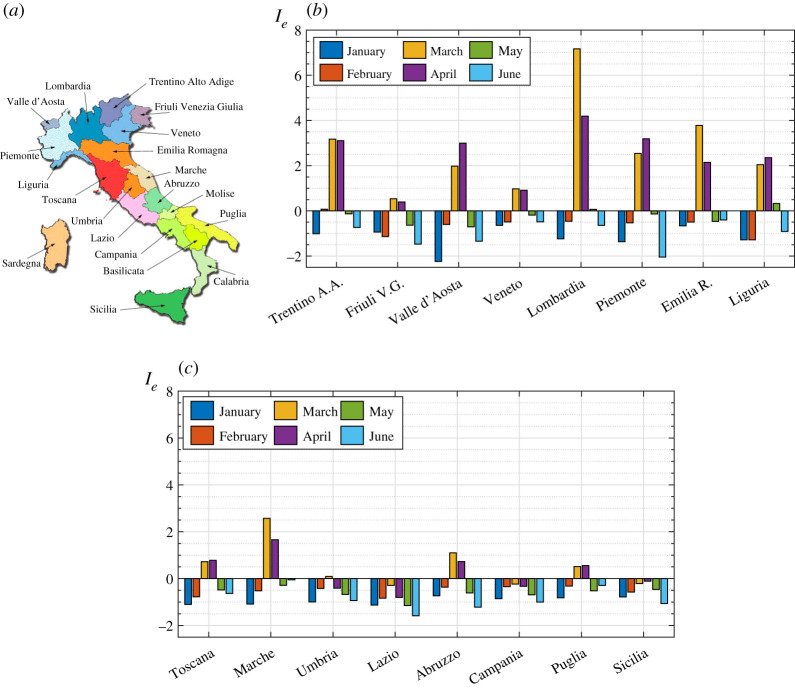
(*a*) Geographic location of the various Italian regions under investigation; (*b*,*c*) values of the *relative index of epidemic* *I*_*e*_ from January 2020 to June 2020 in the northern (*b*) and central and southern (*c*) regions of Italy. Pre-COVID-19 conditions for comparison refer to the average mortality in the same weeks of previous years (2015–2019). (Online version in colour.)

For the 16 considered regions, the values of the measured probability of death *P*^*D*^(45, 1), the calibrated coefficients *γ*_1_ and *γ*_2_ and the calculated quantities *Q*_0_ and *Q*_1_ are recorded in table 2 in the electronic supplementary material. The *relative index of epidemic* *I*_*e*_ has been calculated from (2.8) and the results for the months from January to June are shown as the histograms in figure 2*b*,*c*.

The mortality rates collected in the period January–June 2020 allow the analysis of the whole COVID-19 first wave in Italy. The peak of the epidemic in most regions was reached in March and in April in a few regions (Valle d’Aosta, Piemonte, Liguria). The most infected region was Lombardia followed, with significantly lower numbers, by Emilia Romagna. The epidemic was milder in central Italy, except Marche, where *I*_*e*_ in March and April was much higher than in Toscana and Umbria. South of Lazio the infection was very limited. This is probably because here the epidemic developed later than in the north, so that the lockdown countermeasures, simultaneously imposed on the whole national territory, were more effective. It is quite remarkable that in Lazio, Campania and Sicilia the index *I*_*e*_ never reached positive values and that it was negative for all the considered regions in January, and for most of them in February. Indeed, it should be recalled that, before the spread of COVID-19, the year 2020 was characterized by an unusually low mortality. Recall that *I*_*e*_ only provides a comparative measure: the theory shows that configuration ‘1’ was characterized by a severity of epidemic lower than configuration ‘0’ in that period of observation. Furthermore, it is interesting to notice that in June, and for many regions in May, *I*_*e*_ becomes even more negative than in January–February 2020. This may be attributed to the fact that the long national lockdown limited the strength of the epidemic also with respect to sources other than COVID-19.

**Figure 3.**
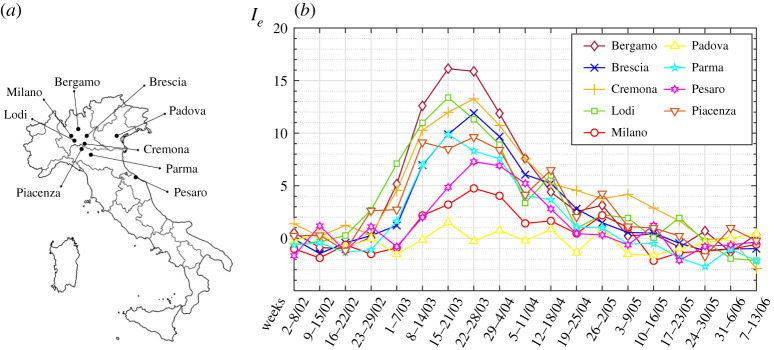
(*a*) Geographic location of the capital city of the considered Italian provinces and of Milano city. (*b*) Variation in the index of epidemic *I*_*e*_ per week, from 2 February to 13 June 2020. Raw mortality data released by ISTAT. Pre-COVID-19 conditions of comparison refer to the average mortality in the same weeks of previous years (2015–2019). (Online version in colour.)

It should be recalled, however, that the epidemic focused on restricted outbreaks within each region. Hence, we now calculate *I*_*e*_ in smaller representative territories, included in the nine Italian provinces shown in figure 3*a*. The calculations are made on a weekly basis from 2 February until 13 June, using the raw mortality data released by ISTAT and available at https://www.istat.it/it/archivio and http://demo.istat.it for the years 2015–2020. The procedure is the same as that used for the regions. The extension of the territories investigated and how the data have been processed are presented in detail in §3b. Values of *I*_*e*_ are plotted against time in figure 3*b*.

Although the first official contagions were recorded on 21–22 February and taking into account the incubation time (about two weeks) and the course of the disease, the index in Lodi, the first infected province, was already high (*I*_*e*_ ≃ 2.681) in the week 23–29 February. It increased one week later (*I*_*e*_ ≃ 7.098), also affecting neighbouring Cremona, Piacenza and Brescia. However, it grew even more in Bergamo (*I*_*e*_ ≃ 5.154), although Bergamo does not border Lodi. Even in Pesaro-Urbino, which is 360 km away from Lodi, *I*_*e*_ quickly reached very high values. Presumably, independent outbreaks developed here and in Bergamo.

Recall that the Italian government imposed the national lockdown on 9 March, but, on 24 February, 10 municipalities in the province of Lodi and one in Padova had already been preventively quarantined. This justifies why *I*_*e*_ decreased first in Lodi, after the peak in the third week of March (*I*_*e*_ ≃ 13.383), but it continued to increase in Brescia and Cremona, while in Bergamo the value reached by the index remained almost unchanged between the third and fourth weeks of March. Furthermore, it is certainly of interest to notice the different slope of the ascending phase of *I*_*e*_ between the provinces of Lodi and Bergamo. The restrictive measures imposed on Lodi nearly two weeks earlier than in Bergamo had a noteworthy effect on the growth rate of *I*_*e*_. In Bergamo *I*_*e*_ exceeded the Lodi peak, achieving a dramatic value of *I*_*e*_ ≃ 16.139. Padova in Veneto was officially considered to be one of the first heavily infected provinces, but this is not confirmed here, probably because the localized outbreak was promptly quarantined. Indeed Veneto is one of the less infected regions in the north, as a consequence of the very effective countermeasure promptly organized by the local authorities. Milano city had been officially considered highly critical, but the virus developed later there than in the other provinces of Lombardia, and this is why the national lockdown prevented the index from reaching very high values.

## 3. Methods

### (a) The statistical theory

The starting point for the theory is the renormalization of the real age *A*_*r*_ to the nominal age *A*_*n*_ through *A*_*n*_ = *F*(*A*_*r*_), to be considered valid on average within a territory reputed to be homogeneous. A particular type of renormalization law is that in (2.1).

The level of ‘human damage’ that an individual can develop is supposed to be measured by the variable *δ*. The model could be readily generalized by introducing additional parameters and following the same rationale, but this is postponed to further studies.

A statistical law *à la* Pareto is postulated, of the type

3.1 $$p_{\Delta A_{n}}(\delta )=C\;\delta^{-\alpha },\;\text{with}\;\alpha >1.$$

This represents the probability density function for the development of a level of human damage *δ*, with reference to the renormalization length scale for human ageing, indicated by the single life-segment Δ*A*_*n*_. This law should universally hold for every person, irrespective of race, genetics, quality of environment and life and gender, because the distinction in these terms is accounted for through the renormalization *A*_*n*_ = *F*(*A*_*r*_).

Since the power-law function diverges for *δ* → 0, it is customary to prescribe a normalization criterion. Hence, we assume that a physiological level of human damage *δ*_min_ is always present in each nominal life-segment. This represents the physiological minimal degradation due to ageing: a person of nominal life *A*_*n*_ will develop at least a level of damage equal to *A*_*n*_/Δ*A*_*n*_ · *δ*_min_, otherwise it would be theoretically possible to live forever. We can then write

3.2 $$\begin{array}{rl} & \int_{\delta_{\text{min}}}^{\infty }p_{\Delta A_{n}}(\delta )\;\text{d}\delta =\int_{\delta_{\text{min}}}^{\infty }C\;\delta^{-\alpha }\;\text{d}\delta =1\\ & \Rightarrow C=\frac{\alpha -1}{{\left(\delta_{\text{min}}\right)}^{-\alpha +1}}\;, \end{array}$$

so that, for *δ* ≥ *δ*_min_, (3.1) becomes

3.3 $$p_{\Delta A_{n}}(\delta )=\frac{\alpha -1}{\delta_{\text{min}}}{\left(\frac{\delta }{\delta_{\text{min}}}\right)}^{-\alpha }.$$

The probability of developing a level of damage more severe than or equal to *δ* ≥ *δ*_min,Δ*t*_ in Δ*A*_*n*_ reads

3.4 $$P_{\Delta A_{n}}^{\geq }(\delta )=\int_{\delta }^{\infty }p_{\Delta A_{n}}(\delta )\text{d}\delta ={\left(\frac{\delta }{\delta_{\text{min}}}\right)}^{1-\alpha },$$

which can be rearranged in the form

3.5 $$P_{\Delta A_{n}}^{\geq }(\delta )={\left(\frac{\delta }{\delta_{\text{min}}}\right)}^{1-\alpha }=\Delta A_{n}{\left(\frac{\delta /\delta_{\text{min}}}{\eta }\right)}^{1-\alpha },$$

where

3.6 $$\eta =\frac{1}{{\left(\Delta A_{n}\right)}^{1/(\alpha -1)}}.$$

Therefore, the probability of developing a damage level lower than *δ* is $P_{\Delta A_{n}}^{<}(\delta )=1-P_{\Delta A_{n}}^{\geq }(\delta )$.

We treat the ‘development of damage in one nominal life-segment’ as an event that compounds statistically with the same event occurring in other life-segments. In the whole nominal life *A*_*n*_, composed of *A*_*n*_/Δ*A*_*n*_ nominal life-segments, the damage level ≥*δ* does not develop if this is true in all the segments. Hence, the probability $P_{A_{n}}^{<}(\delta )$ of finding a damage level less than *δ* in *A*_*n*_ coincides with the product of the probabilities of having the same property in all the Δ*A*_*n*_, i.e.

3.7 $$P_{A_{n}}^{<}(\delta )={\left[1-\Delta A_{n}{\left(\frac{\delta /\delta_{\text{min}}}{\eta }\right)}^{1-\alpha }\right]}^{A_{n}/\Delta A_{n}}.$$

This expression can be simplified under the hypothesis that Δ*A*_*n*_/*A*_*n*_ ≪ 1. Taking the limit as Δ*A*_*n*_/*A*_*n*_ → 0 and observing that lim _*ϵ*→0_ [1 + *a* *ϵ*]^1/*ϵ*^ = exp [*a*], one can write

3.8 $$P_{A_{n}}^{<}(\delta )=\exp\left[-A_{n}{\left(\frac{\delta /\delta_{\text{min}}}{\eta }\right)}^{1-\alpha }\right],$$

or, recalling (3.6),

3.9 $$P_{A_{n}}^{<}(\delta )=\exp\left[-\frac{A_{n}}{\Delta A_{n}}{\left(\frac{\delta }{\delta_{\text{min}}}\right)}^{1-\alpha }\right].$$

Observe that, in this expression, only the ratios between dimensionally homogeneous quantities are present. Of course, (3.9) is to be considered as an approximation of (3.7) when *A*_*n*_/Δ*A*_*n*_ becomes very large. Hence, one has that $P_{A_{n}}^{<}(\delta )≃0$ when *δ*/*δ*_min_ = 1, and $P_{A_{n}}^{<}(\delta )\to 1$ for *δ*/*δ*_min_ → +∞ since *α* > 1.

Therefore, the probability $P_{A_{n}}^{\geq }(\delta )=1-P_{A_{n}}^{<}(\delta )$ of finding in *A*_*n*_ a level of damage ≥*δ* turns out to be that expressed by (2.2).

The force of the epidemic is supposed homogeneous in the considered region and is measured by one parameter *σ*. This again is a simplification, but a more detailed characterization can be obtained by considering *σ* as a function of space (different values in diverse locations).

The event ‘death’ is determined according to a *death criterion* represented by a function G(⋅,⋅,⋅): the event occurs when

3.10 $$G(\delta /\delta_{\text{min}},\sigma ,A_{n})-K_{Ic}=0.$$

Likewise, the ‘safe’ domain is defined by $G(\delta ,\sigma ,A_{n})-K_{Ic}<0$. Given *A*_*n*_, the *critical* level of damage *δ*/*δ*_min_, beyond which death occurs, is associated with the level of epidemic *σ* through a function *δ*/*δ*_min_ = *g*(*σ*, *A*_*n*_), implicitly defined by (3.10), such that

3.11 $$G(g(\sigma ,A_{n}),\sigma ,A_{n})-K_{Ic}=0.$$

It is reasonable to require that *g*( · , *A*_*n*_) is *monotone decreasing* because, for any *A*_*n*_, the higher the force of the epidemic, the lower the level of human damage leading to death. Moreover, one expects that when *σ* → 0 it takes a level of damage *δ*/*δ*_min_ → ∞ to cause death, hence the further condition lim _*σ*→0_*g*(*σ*, *A*_*n*_) = +∞.

Because of these properties, the *probability of death* *P*^*D*^(*A*_*n*_, *σ*) in the epidemic *σ* at the nominal age *A*_*n*_ coincides with the probability of developing a level of damage *δ*/*δ*_min_ = *g*(*σ*, *A*_*n*_). Substituting in (2.2), one obtains

3.12 $$\begin{array}{rl} P^{D}(A_{n},\sigma ) & =P_{A_{n}}^{\geq }(g(\sigma ,A_{n}))\\ & =1-\exp\left[-\frac{A_{n}}{\Delta A_{n}}{\left(\frac{1}{g(\sigma ,A_{n})}\right)}^{\alpha -1}\right]. \end{array}$$

In the simplest case, dropping the dependence of *δ*/*δ*_min_ = *g*(*σ*, *A*_*n*_) on *A*_*n*_, one can consider the power law of (2.3), which satisfies the required monotonicity and asymptotic properties. This is defined by the variables *Y*, *σ* and the parameter *K*_*Ic*_, whose role has been discussed in §2a.

Therefore, (3.12) simplifies into

3.13 $$P^{D}(A_{n},\sigma )=1-\exp\left[-\frac{A_{n}}{\Delta A_{n}}{\left(\frac{\sigma }{\eta_{0}}\right)}^{m}\right],$$

which is a two-parameter Weibull distribution with shape parameter *m* and scale parameter *η*_0_, independent of *σ*, given by

3.14 $$m=(\alpha -1)\beta ,\;\eta_{0}=K_{Ic}/Y.$$

In terms of real-life *A*_*r*_, the renormalization *A*_*n*_ = *F*(*A*_*r*_) provides the expression of (2.4).

There are affinities with the statistical micro-mechanically motivated models describing the population of macroscopic strengths of brittle materials. As pursued in [30], the starting point is a statistical characterization *à la* Pareto of the size *δ* of microcracks in a specimen [32]. LEFM [26] prescribes that cracks propagate when *Yσδ*^1/2^ − *K*_*Ic*_ = 0, where the first term is the stress intensity factor (SIF), *σ* is the nominal applied stress, $Y=2.24/\sqrt{\pi }$ for semicircular thumbnail cracks of radius *δ* and *K*_*Ic*_ is the critical SIF, characteristic of the material. This is why the expression of (2.3) is an ‘LEFM-like’ death criterion, even if the coefficient *β* differs from *β* = 2. In this analogy, the force of the epidemic *σ* represents the applied stress and the nominal life *A*_*n*_ the size of the material specimen [27].

### (b) Analysis of mortality data in Italy

**Figure 4.**
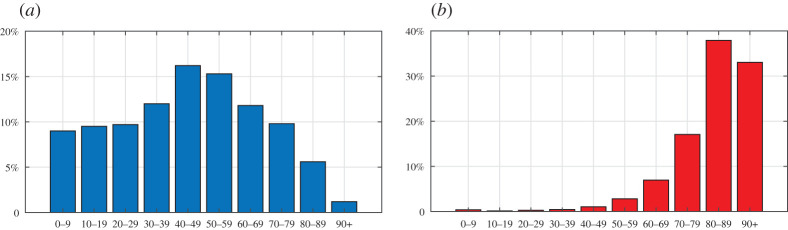
Lombardia, solar year 2018. Percentages according to age of (*a*) the living population and (*b*) the deceased people. (Online version in colour.)

The theory has been applied to the quantitative analysis of the first wave of COVID-19 that affected Italy in the first half of 2020. We analyse 16 regions, considered boxes with homogeneous mortality, distributions of the population and deaths by age. This assumption provides an averaged view, within the same region, of the differences in genetic heritage, quality of environment and life and efficiency of the health system. No differentiations are made between men and women, although a diverse mortality by gender has been recorded. We start by evaluating configuration ‘0’ (pre-COVID-19), chosen to be the period 2015–2019. The ISTAT published mortality data for many Italian municipalities, available online at https://www.istat.it/it/archivio, sorted by age on a daily basis. An example of the demographic structure and mortality data furnished by ISTAT is shown in figure 4 with reference to Lombardia in 2018. It can be seen in figure 4*b* that the datum for the age group 0–9 is higher than that for the 10–19 and 20–29 age groups, presumably because of deaths in the first year of life. This explains why the young age on the left-hand side of figure 1*a* has an increased severity.

The considered municipalities cover most of the population in each region, as indicated in table 1 in the electronic supplementary material.

For any two solar ages *A*_*r*,1_ and *A*_*r*,2_, (2.4) provides the scaling law

3.15 $$\frac{F(A_{r,1})}{F(A_{r,2})}=\frac{\ln[1-P^{D}(A_{r,1},\sigma )]}{\ln[1-P^{D}(A_{r,2},\sigma )]}.$$

**Figure 5.**
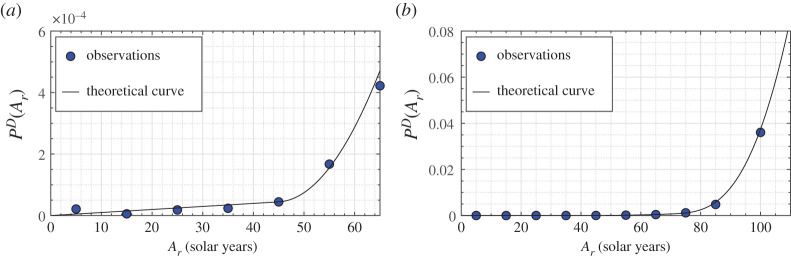
Region of Lombardia in the configuration ‘0’ (pre-COVID-19) condition of March (average of 2015–2019). Points represent the observed probability of death *P*^*D*^ (*A*_*r*_, *σ*) in the month, as a function of real age *A*_*r*_ measured in the number of solar years, while the curve indicates the theoretical prediction. (*a*) Left-hand-side portion for 0 ≤ *A*_*r*_ ≤ 65 solar years; (*b*) right-hand-side portion for *A*_*r*_ ≥ 65 solar years. The same correlation has been constructed for all other considered regions, provinces and for Milano city, by using the demographic statistics published by ISTAT (http://demo.istat.it/). (Online version in colour.)

We start by calibrating the renormalization law *A*_*n*_ = *F*(*A*_*r*_) of the form (2.1). The choice of the ages 45 and 70 as the set points of the renormalization resulted from an analysis of the mortality rates in all 16 considered regions in configuration ‘0’ and from the requirement that the experimental points be aligned in the epidemic Weibull plane, according to the theory. Since *A*_*n*_ = *A*_*r*_ for *A*_*r*_ ≤ 45 solar years, one can fix a point in this range (we fixed *A*_*r*,2_ = 45 solar years) and calculate the coefficients *γ*_1_ and *γ*_2_ by best fitting of the rescaling law of (3.15) with experimental points. The results for the 16 regions are summarized in table 1 in the electronic supplementary material. Notice that, among the various regions, the difference between the values reached by the parameters *γ*_1_ and *γ*_2_ is small. The agreement is in general excellent, as demonstrated by the graphs for Lombardia reported in figure 5. In general, the choice of the set points at 45 and 70 solar years of age provides good results in all the considered regions. We note that the probabilities of death associated with ages lower than 40 years are not optimally fitted by the theoretical curve, but this is due to the fact that they are strongly sensitive to small variations in the number of dead and that deaths at young ages often occur from non-epidemic causes (road accidents, drugs, homicides). In particular, the probability of death in the range 0–10 years is always higher than the theoretical prediction, but this is certainly influenced by the number of deaths at birth, which represents a special category. In general, our model does not account for deaths due to factors that are not associated with the natural degradation of the human body (human damage) and the consequent potential ability to develop a pathology.

The theory assumes that the renormalization function (2.1) is not affected by the level of epidemic, i.e. the function G in equation (3.10) is independent of *Yσ*, and hence the parameters *γ*_1_ and *γ*_2_ are the same in configurations ‘0’ and ‘1’. A confirmation of this is that in the epidemic Weibull plane the experimental points are also aligned in configuration ‘1’, according to equation (2.5). Of interest is the intercept of the interpolation line with the ordinate axis, referred to as *Q* in (2.6).

The value of *Q* in configuration ‘0’ (years 2015–2019), referred to as *Q*_0_, is obtained graphically from the line that best fits the measured points. To illustrate, the data for Lombardia in March, plotted in figure 1*a*, provide *Q*_0_ = −13.877. In configuration ‘1’ (March 2020), with an analogous graphical construction shown in figure 1*b*, one finds the value *Q*_1_ = −12.949. Hence, the *epidemic ratio*, defined in (2.7), is *r* = *Q*_0_/*Q*_1_ = 1.072. From the graph in figure 1*b* it is also possible to appreciate how the renormalization parameters of (2.1), calibrated for configuration ‘0’, provide a good fit for configuration ‘1’. Passing to the *relative index of epidemic* *I*_*e*_ = 100 · (*r* − 1), as per equation (2.8), one finds in Lombardia the value *I*_*e*_ = 100 · (1.072 − 1) = 7.2.

**Figure 6.**
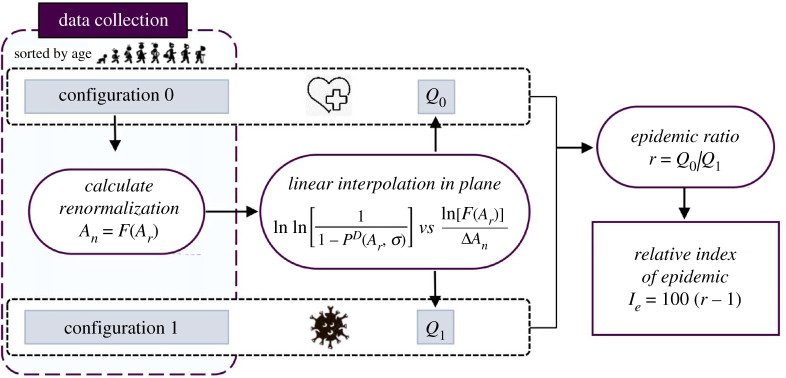
Schematic flowchart of the procedure used to calculate the relative index of epidemic. Mortality data in the configuration ‘1’ (COVID-19 epidemic, period in 2020) and configuration ‘0’ (corresponding months in previous years) conditions, sorted by age, are compared. The renormalization *A*_*n*_ = *F*(*A*_*r*_), where *A*_*n*_ and *A*_*r*_ are the nominal and real ages, respectively, is calibrated from configuration ‘0’. The best-fit interpolation in the plane lnln{[1 − *P*^*D*^(*A*_*r*_, *σ*)]^−1^} versus ln[*F*(*A*_*r*_)/Δ*A*_*n*_] of the measured points with the line predicted by the theory provides the values *Q*_0_ (configuration ‘0’) and *Q*_1_ (configuration ‘1’). The *epidemic ratio* *r* = *Q*_0_/*Q*_1_ and the *relative index of epidemic* *I*_*e*_ = 100(*r* − 1) are then calculated. (Online version in colour.)

The procedure is summarized in the flowchart in figure 6. The calculated *I*_*e*_ in each of the 16 considered regions has been shown in the histograms in figure 2*b* for the various monthly periods of observation. The numerical values of *I*_*e*_, together with the other calculated relevant parameters, are recorded in table 2 in the electronic supplementary material.

Moving on to the analysis of the provinces, indicated in figure 3*a*, their population has been obtained by grouping the municipalities according to their location, considering the mortality data week by week. As indicated in table 3 in the electronic supplementary material, the samples cover more than 95% of the population in each province, apart from Padova, where only 82.26% of the population is considered because data are not available. The city of Milano, which is densely populated, has been analysed independently from the other municipalities in its province. For each considered week, the probability of death sorted by age in configuration ‘0’ has been calculated with reference to the average number of deaths that occurred in the corresponding weeks in previous years. The numbers of deaths per day, sorted by age, are available at www.istat.it/it/archivio, while the total population at the beginning of each month is available at www.demo.istat.it, together with its categorization by age on a yearly basis. Since ISTAT provides the demographic structures of the provinces only until the year 2019, we have assumed that, in configuration ‘1’, population by age is the same as in 2019, and the total population has been estimated according to the demographic trend.

Following the same procedure used for the regions, the results are recorded in table 4 in the electronic supplementary material for configurations ‘0’ and ‘1’, calculated on a weekly period of observation. Consistent with the results of the regions, the coefficients *γ*_1_, *γ*_2_ and *Q*_0_ vary only slightly among the provinces during the observation weeks. With respect to the regions, there is a difference in the data analysis in the fact that, in configurations ‘0’, we have assumed that the probability of death *P*^*D*^(*A*_*r*,2_) at *A*_*r*,2_ = 45 solar years of age is the same for all provinces and equal to the probability of death in the whole national territory corresponding to the age range 40–50 years in the third week of March in the period 2015–2019. The reason for this is that the probabilities of death in the classes corresponding to the younger ages are strongly affected by small deviations in mortality, especially if the number of inhabitants is small, as in provinces with respect to the regions. If we had considered as *P*^*D*^(*A*_*r*,2_) the observed value in the province, there would have been a significant uncertainty in the evaluations of the index, especially for the less populated territories. In fact, mortality among the young may be strongly affected by random sources, such as car accidents, whose effect is greater when the size of the set under consideration is smaller.^2^ The national datum appears more reliable for deaths from natural causes.

The values of *Q*_0_ and *Q*_1_, reported in the same table, have been graphically estimated and the resulting values of *I*_*e*_ have been plotted against time in figure 3*b*.

## 4. Discussion

**Figure 7.**
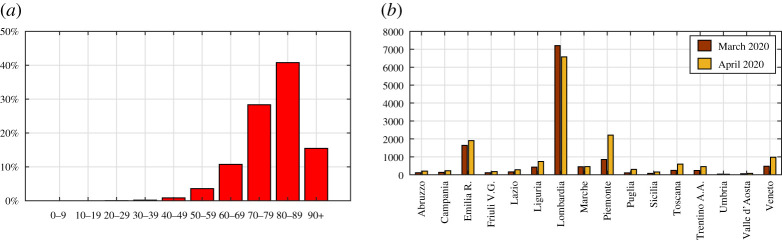
(*a*) Distribution by age of deaths attributed to COVID-19 in Italy, published by the Italian National Institute of Health (ISS) on 4 May 2020 (https://www.epicentro.iss.it/coronavirus). (*b*) Number of deaths attributed to COVID-19 in March and April 2020, sorted by region, as provided by the Civil Protection Department of Italy on 30 April 2020 (http://www.protezionecivile.gov.it/home). (Online version in colour.)

Most models consider the evolution of epidemics on the basis of the number of infected people and/or deaths from contagion [33–37]. However, this number can be correctly estimated if and only if tests are performed on at least a representative sample [38,39], which is not always possible. Figure 7*a* shows the age distribution of deaths in Italy attributed to COVID-19 up to 4 May 2020, shortly after the epidemic peak, as provided by the Italian National Institute of Health (ISS). Comparison with figure 4*b*, which shows the distribution for the year 2018 in Lombardia, indicates a surprisingly low mortality rate in people over the age of 90. Most likely, positivity to COVID-19 had not been tested in all the dead [40]. Moreover, figure 7*b* records the number of deaths attributed to COVID-19 in the months of March and April 2020, sorted by region, as provided by the Civil Protection Department of Italy (http://www.protezionecivile.gov.it/home). In the northern regions, the number of deaths in March–April 2020 officially attributed to COVID-19 is 24 130. The total number of deaths in the same regions and in the same months, derived from mortality tables released by ISTAT (https://www.istat.it/it/archivio), is 90 984 in 2020, while the average number in the years 2015–2019 is 48 985. The difference is 41 999, roughly twice the official number of deaths from COVID-19. This example raises questions about the ability of national institutions to identify all deaths attributable to a new virus such as COVID-19, especially in the acute phase of the epidemic and, therefore, underlines the importance of monitoring the evolution of an epidemic through objective parameters, such as the raw mortality tables.

**Figure 8.**
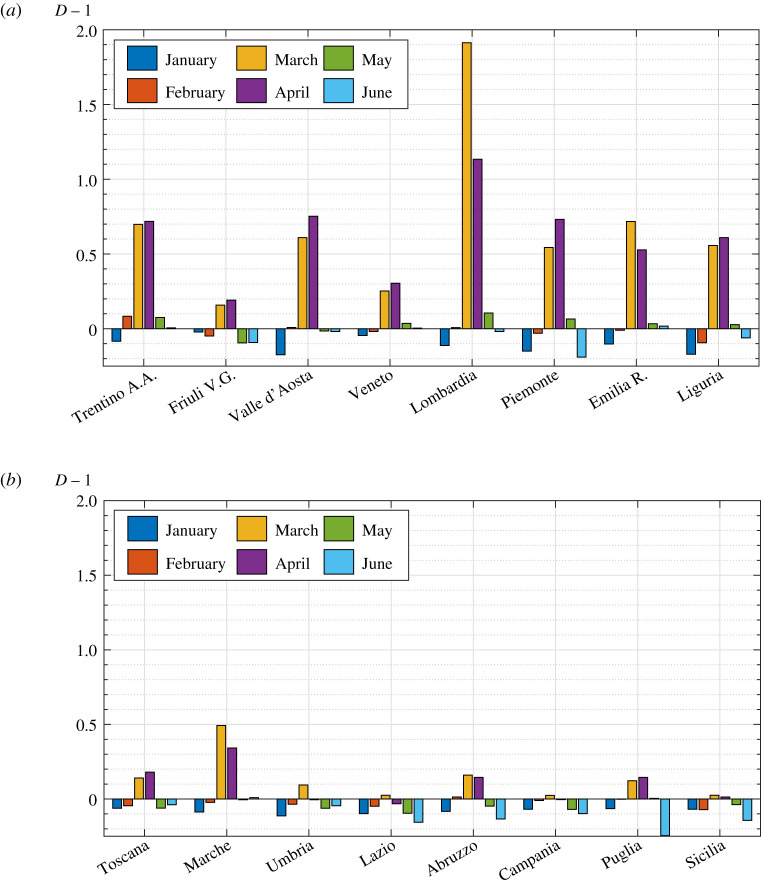
Values of *D* − 1 from January to June 2020 in the (*a*) northern and (*b*) central and southern regions of Italy. The number of deaths in the months of 2020 has been compared with the average number in the same months of the years 2015–2019. (Online version in colour.)

Other criteria have been proposed to estimate the strength of an epidemic. With particular reference to raw mortality tables, it is customary to consider as a representative indicator the *differential mortality* *D*, defined as the ratio between the number of deaths in the epidemic condition and those recorded in previous years. We consider *D* − 1 instead of *D*, so that the null value will indicate equality between the pre- and post-COVID-19 conditions, as it is for *I*_*e*_. Figure 8*a*,*b* reports the value of *D* − 1 in January–June 2020 in all the considered regions, and represents the counterpart of the histograms for *I*_*e*_ shown in figure 2*b*,*c*. The differential mortality has been calculated by comparing the number of deaths in the months of 2020 with the average number in the same months of the years 2015–2019. The numerical values for March 2020 are recorded in table 5 in the electronic supplementary material. Of course *I*_*e*_ and *D* − 1 cannot be directly compared in absolute terms, but their relative values in the various regions can be appreciated.

The trends of *D* − 1 and *I*_*e*_ are quite similar in the various regions, but there are conceptual differences. Compare, for example, Trentino Alto Adige with Liguria or Valle d’Aosta: the differences in terms of *D* − 1 in the acute phase of the epidemic are small, but figure 2*b* shows that this is not true^3^ in terms of *I*_*e*_. The difference is due to the different demographic structure. Trentino Alto Adige is a relatively ‘young’ region, where about 58% of the population is under the age of 50, while this percentage drops to 53% in Valle d’Aosta and to 48% in Liguria. The proposed theory makes a distinction between deaths in older people and those in younger people, associated with the greater aptitude of the former to develop life-threatening diseases during epidemics. This means that a greater variation in the number of deaths in older people is recognized as a different sign of the effects of an epidemic with respect to an equal variation in the young. According to this rationale, the differential mortality index in Trentino would lead, because of its young demographic structure, to an underestimation of the ‘strength’ of the epidemic. The same observation can be made also for other regions. Moreover, note that *I*_*e*_ never reaches positive values in Lazio, Campania, and Sicilia, whereas *D* − 1 in March is positive, albeit slightly. This is because the increase in deaths in these regions was higher among the younger than among the older population. In conclusion, the *differential mortality* indicator can certainly provide useful information about the effect of an epidemic, but it cannot consider the distribution of deaths by age and the demographic structure of the population, which should instead play a significant role.

Recall that the *epidemic ratio* *r*, and hence the *relative index of epidemic* *I*_*e*_, depends on the variables *σ*, *Y* and *β*, which respectively take into account the force (lethality) of the virus, its level of spread and our ability to cure the disease, as per equation (2.7). It is reasonable to assume that *β* and *σ* have almost the same values in all Italian regions. Hence, the observed differences in terms of *I*_*e*_ should be attributed to the level of spread *Y*, which varies over time in the various regions owing to the spread of the virus and can be constrained by institutional actions such as imposed social distancing, reduced mobility, use of face masks and lockdown. On the other hand, an increase in *β* can only derive from an increased efficiency of the health system, the discovery of an effective cure or the administration of a vaccine, while a decrease in *β* can be interpreted as a negative effect being induced, e.g. by exceeding the capacity of intensive care units.

In order to analyse how the *relative index of epidemic* is influenced by a variation in *β*, theoretically compare two different configurations 1, say ‘1a’ and ‘1b’, at the same time of observation. From equation (2.7) one derives that the corresponding epidemic ratios *r*_*a*_ and *r*_*b*_ satisfy the scaling law

4.1 $$\frac{r_{b}}{r_{a}}=\frac{\beta_{1a}}{\beta_{1b}}\;\frac{\ln[K_{Ic}/(Y_{1a}\;\sigma_{1a})]}{\ln[K_{Ic}/(Y_{1b}\;\sigma_{1b})]},$$

where *β*_1*a*_, *Y*_1*a*_, *σ*_1*a*_ and *β*_1*b*_, *Y*_1*b*_, *σ*_1*b*_ are the model parameters in the configurations ‘1a’ and ‘1b’, respectively, whereas *K*_*Ic*_ is clearly invariant.

If the spread and the force of the epidemic is the same for the two configurations, i.e.*Y*_1*a*_ = *Y*_1*b*_ and *σ*_1*a*_ = *σ*_1*b*_, then (4.1) provides

4.2 $$r_{b}=\frac{\beta_{1a}}{\beta_{1b}}\;r_{a}.$$

**Figure 9.**
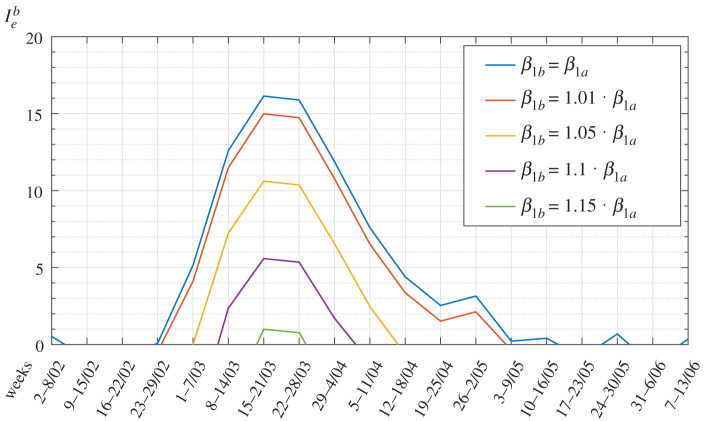
First wave of COVID-19 in *Bergamo*. Expected variation of the *relative index of epidemic* with the parameter *β*. (Online version in colour.)

Consider, for example, the first wave of the epidemic in Bergamo, the most affected Italian province, and let $I_{e}^{a}=100(r_{a}-1)$ denote the measured actual index of epidemic. Figure 9 shows the index $I_{e}^{b}=100(r_{b}-1)$ corresponding to the case in which *Yσ* is left unchanged and *β* is varied. Observe that even a very small increase in *β* provides a strong effect.

In order to analyse the effects of a variation in *Y*, the two configurations ‘1a’ and ‘1b’ to be compared are defined by *σ*_1*a*_ = *σ*_1*b*_ = *σ* and *β*_1*a*_ = *β*_1*b*_ = *β*. Setting *ψ* = *Y*_1*a*_/*Y*_1*b*_, from (4.1) one obtains

4.3 $$r_{b}=r_{a}\frac{\ln[K_{Ic}/(Y_{1b}\;\sigma )]-\ln\psi }{\ln[K_{Ic}/(Y_{1b}\;\sigma )]}\Rightarrow \psi ={\left(\frac{K_{Ic}}{Y_{1b}\;\sigma }\right)}^{1-(r_{b}/r_{a})}.$$

Our aim now is to evaluate the evolution of the coefficient *Y* during the first wave of the epidemic. Referring again to the case of Bergamo, consider configuration ‘1b’ as the one corresponding to the first week of February (2–8 February) when, according to figure 3, the wave was just about to begin. At this stage, the force of the epidemic was *σ*_1*b*_ = *σ* and its level of spreading *Y*_1*b*_. Consider configurations ‘1a’ corresponding to the successive weeks, for which *σ*_*a*_ = *σ* remains constant but the spreading varies, i.e. *Y*_1*a*_ = *Y*_1*a*_(*t*) = *ψ*(*t*)*Y*_*b*_, in order to emphasize the dependence upon time. Clearly, in the week 2–8 February, one finds *Y*_*a*_ = *Y*_*b*_ and *r*_1*a*_ = *r*_1*b*_, so that *ψ* = 1 from (4.3). Setting *r*_*a*_ = *r*_*a*_(*t*), now a function of time determined from the analysis of the mortality data, *ψ* = *ψ*(*t*), can be determined from (4.3) since the ratio *r*_*b*_/*r*_*a*_(*t*) is known.

Recall from the discussion at the end of §2a that this theory can only compare any two different states. In particular, in (4.3) the number *Y*_1*b*_/*σK*_*Ic*_ represents the term of comparison for the force of the epidemic and its level of spread. Of course, this could be expressed as a function of any other state. For example, referring to configuration ‘0’ defined by the average mortality rates in the first week of February in years before COVID-19, with epidemic values *β*_0_, *Y*_0_ and *σ*_0_, since presumably *β*_0_ = *β*_1*b*_ = *β*, from (2.7) one obtains

4.4 $$r_{b}=\frac{\ln[K_{Ic}/(Y_{0}\;\sigma_{0})]}{\ln[K_{Ic}/(Y_{1b}\;\sigma_{1b})]}\Rightarrow [K_{Ic}/(Y_{1b}\;\sigma_{1b})]={\left[K_{Ic}/(Y_{0}\;\sigma_{0})\right]}^{1/r_{b}}.$$

**Figure 10.**
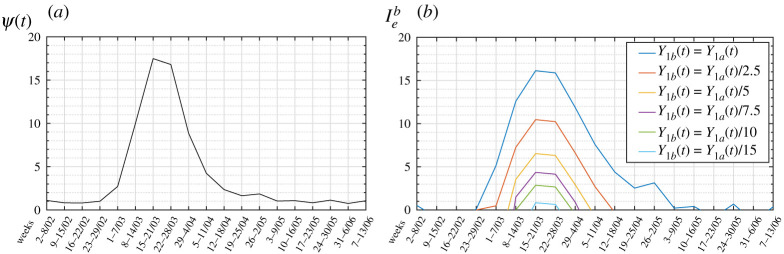
First wave of COVID-19 in Bergamo. (*a*) Calculated function *ψ*(*t*) for *Y*_0_ *σ*_0_/*K*_*Ic*_ = 10^−9^; (*b*) expected variation in *I*_*e*_ with a proportional reduction in the level of spread *Y*(*t*). (Online version in colour.)

Certainly *Y*_0_*σ*_0_/*K*_*Ic*_ is a small number since it refers to a pre-COVID-19 condition: this can be chosen arbitrarily as the unit of measurement for the epidemic level. Setting *Y*_0_ *σ*_0_/*K*_*Ic*_ = 10^−9^, from (4.4) and (4.3) one obtains the function *ψ*(*t*) for Bergamo on a weekly basis, from 2 February to 13 June. Its plot is represented in figure 10*a*.

Observe that *ψ*(*t*) is increasing up to the peak in the week of 15–21 March, and then decreases. This is an evident effect of the restrictive countermeasures that were organized in Bergamo; otherwise, the spreading would have continued to increase.

What would have happened if stricter countermeasures had been organized in order to limit further the level of spread? Consider configuration ‘1a’ to be the same as before, for which *Y*_1*a*_(*t*) has just been calculated through the function *ψ*(*t*). Configuration ‘1b’ now corresponds to a purely theoretical state in which the values of *σ* and *β* are left unchanged, whereas the level of spread is *Y*_1*b*_(*t*) = *Y*_1*a*_(*t*)/*k*, where *k* ≥ 1 is a constant. In equation (4.3) now *ψ* = *ψ*(*t*) is substituted by *ψ*(*t*)/*k*, *r*_*a*_ = *r*_*a*_(*t*) is known, and one finds *r*_*b*_ = *r*_*b*_(*t*) and, therefore, $I_{e}^{b}=100\;(r_{b}-1)$. The corresponding plots are represented in figure 10*b* for various values of *k*. While comparing figures 9 and 10*b*, observe that a small variation in *β* has a very strong effect on the spread of the epidemic, while the dependence upon *Y* is much milder. This is not surprising, because the discovery of an effective cure or an improvement in the capacity of intensive care units represent important step-changes, whereas limiting the epidemic with countermeasures such as social distancing requires a great effort to obtain limited results.

Of course, further research is still needed. This theory defines a measure of the strength of the epidemic as a function of the variables *Y*, *σ*, *β*, *K*_*Ic*_, but does not provide their specific expressions as a function, for example, of the capacity of the health system and the type and duration of the restrictive countermeasures organized to limit the spread. What is still needed is a precise definition of the specific mathematical expression for these variables as a function of other more specialized state variables. To this aim, reference should be made to models that can describe the kinetics of the epidemic from balance equations, as in the SIR approach. Indeed, the proposed theory is by no means an alternative to models of this kind, but complementary to them.

In particular, we conjecture that the variable *Y*, and hence the value of *I*_*e*_, is strictly associated with the *epidemic reproduction number* *R*_*t*_. Of course, *R*_*t*_ and *I*_*e*_ refer to events shifted in time: the former indicates contagions, while the latter is found from mortality rates. Hence, *I*_*e*_ shall be compared with values of *R*_*t*_ estimated some time previously, corresponding to the average time between diagnosis and death. In any case, whereas the estimate of *R*_*t*_ requires time to collect and select data^4^ and is strongly influenced by the quality of data [41,42], the estimate of *I*_*e*_ is much more rapid, since it is found from raw mortality. On the other hand, once the correlation between *Y* and *R*_*t*_ is defined, the *relative index of epidemic* might also be used as a tool for verifying the goodness of the estimate of *R*_*t*_.

## 5. Conclusions

A multi-scale statistical theory has been proposed to define a *relative index of epidemic* for the comparative quantification of its potential lethality (strength), with respect to a population observed at different times in any two epidemic configurations. A peculiar feature is the renormalization of the human age by assigning to older people a greater predisposition than younger people to develop a physical degradation (‘human damage’), which favours the onset of pathologies and co-morbidities potentially critical in case of infection. The probability of finding a certain level of human damage in one individual provides the statistical distribution for the risk of death with respect to a yield criterion, which accounts for the force (lethality) of the virus, the level of spread and our capacity to cure the disease, all interpreted through mathematical variables. Remarkably, this rationale conforms to the classical Weibull theory of the statistical strength of brittle structures containing a population of crack-like defects, in correlating the statistical spatial distribution of crack size and shape with the risk of fracture at a prescribed stress level, according to a fracture toughness criterion that determines the onset of catastrophic crack propagation in the structure.

The input for the estimates is represented by raw data on mortality, regardless of the cause of death but sorted by age, collected in various configurations corresponding to different time intervals of observation. As an example, the theory has been applied to the first wave of COVID-19 in Italy (January–June 2020), taking as configurations of comparison the conditions of previous years, certainly characterized by milder forms of epidemics. The theory predicts precise scaling laws for the age of death in the various configurations, which agree very well with the observations in the Italian regions and provinces on a weekly or monthly basis. The proposed index of epidemic, taking into account the demographic structure of the population, weighs the number of deaths according to a risk based on age; therefore, it appears more accurate than other indicators considering the excess mortality rates, which are unable to distinguish deaths from accidental causes. The comparison of the results of our theory and the excess mortality rates reveals similarities at the qualitative level, but also conceptual differences in territories characterized by diverse demographic structures.

An advantage of this theory is that it uses raw mortality data, which are usually considered the least assumption-laden records of the effect of an epidemic, but an extension could be made to other categories, such as the number of people needing intensive care unit beds. The major limitation, at least at the current stage, consists in the lack of a correlation between the parameters of the theory (level of human damage, lethality of the virus, level of spread, capacity of the health system) and the extensive variables on which such parameters depend, such as factors specific to the patient and the virus (co-morbidities, antibody-dependent enhancement, immune history), to the health system (availability of beds, intensive care services, medications, treatments, diagnostic tests, vaccines) and to the control of the spread (social distancing, use of face masks, restrictions, quarantine, lockdown). Certainly, this correlation shall be informed by the existing mathematical models that describe the kinetics of an epidemic, such as the SIR approach and its derivations, for which the present theory is certainly not an alternative but is complementary. A comprehensive multidisciplinary approach is certainly of paramount importance for the management of the epidemic, in particular to make decisions about when, where and to what extent to apply or release restrictive measures, in particular the lockdown which, if prolonged beyond necessity, may cause irreparable damage to the economy of a country.

## Supplementary Material

Supplementary Material

## Footnotes

1 This takes as the input any real number and outputs the same number if this is non-negative and 0 otherwise.

2 For example, one single car accident where five people in their twenties die may strongly alter the death rate in the same interval of age in a province where the number of inhabitants at that age is small, also taking into account that the number of deaths in that category is usually much smaller than that for older people. This effect is somehow ‘diluted’ in a larger population, such as that of a region.

3 Table 5 in the electronic supplementary material indicates that, in March, *D* − 1 is 0.698 in Trentino A.A., 0.557 in Liguria, 0.609 in Valle d’Aosta, whereas the corresponding values for *I*_*e*_ are, respectively, 3.172, 2.046, 1.978.

4 The researchers at the Bruno Kessler Foundation in Trento are in charge of estimating *R*_*t*_ on behalf of the Italian authorities. In their estimations, they only consider symptomatic infected people and disregard people who have contracted the virus abroad. According to the Italian ISS, a period of between 7 and 15 days is necessary to obtain a good estimate of *R*_*t*_. Unfortunately, to the best of our knowledge, at the time of writing the official estimates of *R*_*t*_ have not yet been released for the period considered here. Hence, the comparison between *R*_*t*_ and *I*_*e*_ cannot be done now.

## Data accessibility

Data used in this article are available online at the following official websites of Italian national institutions: https://www.istat.it/it/archivio, http://demo.istat.it/, https://www.epicentro.iss.it/, http://www.protezionecivile.gov.it/home.
