# Supplementary material for: A statistical theory of the strength of epidemics: an application to the Italian COVID-19 case
Source: Proc Math Phys Eng Sci. 2020 Dec 23;476(2244):20200394. doi: 10.1098/rspa.2020.0394 (PMC7776968; doi:10.1098/rspa.2020.0394)
Supplement: Supplementary Material [file rspa20200394supp1.pdf]

A statistical theory of the strength of epidemics. An application to the Italian COVID-19 case

Gabriele PISANO[1] and Gianni ROYER-CARFAGNI *[1,2]

[2]Department of Engineering and Architecture, University of Parma, Parco Area delle Scienze 181/A, I-43100 Parma, Italy

[1]Construction Technologies Institute - Italian National Research Council (ITC-CNR), Viale Lombardia 49, I-20098 San Giuliano Milanese, Milano, Italy

Supplementary Material

This Section contains supplementary materials to the article titled "A statistical theory of the strength of epidemics. An application to the Italian COVID-19 case". The following tables report the numerical values, with reference to the Italian COVID-19 case, which correspond to the data used in the article and the results obtained from their manipulation on the basis of the proposed theory. Charts and graphs reported in the text use these data.

Table 1: Percentage of the total regional population covered by the mortality data in the period 2015-2020, realeased by ISTAT (https://www.istat.it/it/archivio).

**Abruzzo**	**Campania**	**Emilia R.**	**Friuli V.G.**	**Lazio**	**Liguria**	**Lombardia**	**Marche**
93.37%	94.23%	97.07%	94.81%	93.64%	97.80%	98.52%	90.33%
**Piemonte**	**Puglia**	**Sicilia**	**Toscana**	**Trentino A.A.**	**Umbria**	**Valle d'Aosta**	**Veneto**
95.46%	95.88%	90.91%	95.62%	93.53%	95.50%	91.20%	92.25%

*Corresponding author

Email addresses: pisano@itc.cnr.it (Gabriele Pisano), gianni.royer@unipr.it (Gianni Royer-Carfagni)

Table 2: Configuration "0" (average of 2015-2019) and configuration "1" (2020), found by elaborating raw mortality data released by ISTAT in 16 Italian regions. Measured probability of death $P^D(A_{r,2},\sigma)$ at $A_{r,2} = 45$ solar years of age. Parameters γ_1 and γ_2 of the renormalization $A_n = F(A_r)$, as per equation (2.1). Values for $-Q_0$ and $-Q_1$ derived from interpolation of the measured points with the linear trend in the epidemic Weibull plane predicted by the theory. Values of the *epidemic ratio* r and of the corresponding *relative index of epidemic* I_e.

	**Abruzzo**	**Campania**	**Emilia R.**	**Friuli V.G.**	**Lazio**	**Liguria**	**Lombardia**	**Marche**
**Jenuary**								
$P^D(45,1)$	$6.8\cdot10^{-5}$	$8.1\cdot10^{-5}$	$4.9\cdot10^{-5}$	$5.2\cdot10^{-5}$	$6.2\cdot10^{-5}$	$6.1\cdot10^{-4}$	$5.3\cdot10^{-5}$	$5.0\cdot10^{-5}$
γ_1	1.87	1.94	2.00	2.01	1.96	1.98	1.97	1.98
γ_2	2.98	3.02	3.09	3.07	3.05	3.02	3.09	3.10
$-Q_0$	13.398	13.229	13.775	13.700	13.522	13.574	13.667	13.760
$-Q_1$	13.497	13.343	13.867	13.830	13.676	13.750	13.838	13.911
r	0.9927	0.9915	0.9934	0.9906	0.9887	0.9872	0.9876	0.9891
I_e	-0.733	-0.854	-0.663	-0.940	-1.126	-1.280	-1.236	-1.085
**February**								
$P^D(45,1)$	$5.3\cdot10^{-5}$	$7.5\cdot10^{-5}$	$4.8\cdot10^{-5}$	$4.8\cdot10^{-5}$	$5.0\cdot10^{-5}$	$4.6\cdot10^{-5}$	$4.9\cdot10^{-5}$	$4.3\cdot10^{-5}$
γ_1	1.98	1.90	1.94	2.01	1.98	2.02	1.95	2.00
γ_2	2.97	2.97	3.04	3.03	3.05	3.05	3.05	3.08
$-Q_0$	13.673	13.302	13.768	13.755	13.745	13.827	13.754	13.905
$-Q_1$	13.673	13.348	13.837	13.913	13.861	14.002	13.817	13.978
r	0.9964	0.9966	0.9950	0.9886	0.9916	0.9875	0.9954	0.9948
I_e	-0.364	-0.345	-0.499	-1.136	-0.837	-1.250	-0.456	-0.522
**March**								
$P^D(45,1)$	$6.6\cdot10^{-5}$	$7.1\cdot10^{-5}$	$5.1\cdot10^{-5}$	$5.3\cdot10^{-5}$	$5.5\cdot10^{-5}$	$5.3\cdot10^{-5}$	$4.4\cdot10^{-5}$	$5.4\cdot10^{-5}$
γ_1	1.87	1.95	1.93	1.95	1.95	1.98	2.01	1.92
γ_2	2.92	3.00	3.02	3.01	3.02	3.02	3.08	3.02
$-Q_0$	13.46	13.392	13.707	13.663	13.632	13.718	13.877	13.676
$-Q_1$	13.314	13.423	13.208	13.590	13.672	13.443	12.949	13.333
r	1.0110	0.9977	1.0378	1.0054	0.9971	1.0205	1.0717	1.0257
I_e	1.097	-0.231	3.778	0.537	-0.293	2.046	7.167	2.573
**April**								
$P^D(45,1)$	$6.3\cdot10^{-5}$	$7.4\cdot10^{-5}$	$5.4\cdot10^{-5}$	$5.2\cdot10^{-5}$	$4.9\cdot10^{-5}$	$4.3\cdot10^{-5}$	$4.6\cdot10^{-5}$	$5.0\cdot10^{-5}$
γ_1	1.82	1.90	1.88	1.93	1.98	2.01	1.99	1.91
γ_2	2.89	2.95	2.97	2.98	3.02	3.02	3.02	3.01
$-Q_0$	13.471	13.355	13.683	13.715	13.751	13.866	13.850	13.776
$-Q_1$	13.374	13.399	13.396	13.661	13.862	13.547	13.293	13.551
r	1.0073	0.9967	1.0214	1.0040	0.9920	1.0235	1.0419	1.0166
I_e	0.7253	-0.328	2.142	0.395	-0.801	2.355	4.190	1.660
**May**								
$P^D(45,1)$	$5.2\cdot10^{-5}$	$7.0\cdot10^{-5}$	$5.2\cdot10^{-5}$	$3.2\cdot10^{-5}$	$5.7\cdot10^{-5}$	$4.1\cdot10^{-5}$	$4.9\cdot10^{-5}$	$4.3\cdot10^{-5}$
γ_1	1.88	1.92	1.93	2.12	1.93	2.02	1.94	1.98
γ_2	2.94	2.93	2.98	3.10	2.96	3.04	3.00	3.05
$-Q_0$	13.623	13.389	13.735	14.182	13.620	13.916	13.763	13.905
$-Q_1$	13.707	13.482	13.799	14.273	13.778	13.871	13.754	13.945
r	0.9939	0.9931	0.9954	0.9936	0.9885	1.0032	1.0007	0.9971
I_e	-0.613	-0.690	-0.464	-0.638	-1.147	0.324	0.065	-0.287
**June**								
$P^D(45,1)$	$4.0\ 7.1\cdot10^{-5}$	$\cdot10^{-5}$	$5.7\cdot10^{-5}$	$5.8\cdot10^{-5}$	$5.5\cdot10^{-5}$	$6.2\cdot10^{-5}$	$4.9\cdot10^{-5}$	$5.7\cdot10^{-5}$

γ_1	1.99	1.88	1.87	1.89	1.95	1.87	1.94	1.85
γ_2	3.02	2.92	2.93	2.92	2.96	2.92	2.98	2.95
$-Q_0$	13.915	13.381	13.628	13.601	13.683	13.547	13.766	13.645
$-Q_1$	14.087	13.516	13.683	13.804	13.904	13.672	13.855	13.652
r	0.9878	0.9900	0.9960	0.9853	0.9841	0.9909	0.9936	0.9995
I_e	-1.221	-0.999	-0.402	-1.471	-1.589	-0.914	-0.642	-0.051

	**Piemonte**	**Puglia**	**Sicilia**	**Toscana**	**Trentino A.A.**	**Umbria**	**Valle d'Aosta**	**Veneto**
**Jenuary**								
$P^D(45,1)$	$5.9 \cdot 10^{-5}$	$6.8 \cdot 10^{-5}$	$7.4 \cdot 10^{-5}$	$5.7 \cdot 10^{-5}$	$4.9 \cdot 10^{-5}$	$5.1 \cdot 10^{-5}$	$6.4 \cdot 10^{-5}$	$5.4 \cdot 10^{-5}$
γ_1	1.96	1.90	1.91	1.96	2.02	1.95	1.96	1.95
γ_2	3.08	3.03	3.03	3.04	3.05	3.09	3.04	3.07
$-Q_0$	13.575	13.414	13.303	13.611	13.785	13.734	13.478	13.660
$-Q_1$	13.763	13.525	13.408	13.763	13.926	13.872	13.787	13.748
r	0.9863	0.9918	0.9922	0.9890	0.9899	0.9901	0.9776	0.9936
I_e	-1.366	-0.821	-0.783	-1.104	-1.012	-0.995	-2.241	-0.640
**February**								
$P^D(45,1)$	$5.5 \cdot 10^{-5}$	$5.9 \cdot 10^{-5}$	$6.4 \cdot 10^{-5}$	$5.4 \cdot 10^{-5}$	$6.1 \cdot 10^{-5}$	$4.2 \cdot 10^{-5}$	$2.2 \cdot 10^{-5}$	$4.8 \cdot 10^{-5}$
γ_1	1.94	1.86	1.91	1.90	1.81	1.98	2.28	1.94
γ_2	3.04	3.02	3.03	3.01	2.96	3.08	3.30	3.06
$-Q_0$	13.655	13.547	13.452	13.663	13.552	13.926	14.520	13.767
$-Q_1$	13.728	13.590	13.530	13.770	13.542	13.985	14.608	13.835
r	0.9947	0.9968	0.9942	0.9922	1.0007	0.9958	0.9940	0.9951
I_e	-0.532	-0.316	-0.576	-0.777	0.074	-0.422	-0.602	-0.492
**March**								
$P^D(45,1)$	$4.6 \cdot 10^{-5}$	$6.3 \cdot 10^{-5}$	$7.1 \cdot 10^{-5}$	$4.5 \cdot 10^{-5}$	$4.7 \cdot 10^{-5}$	$5.4 \cdot 10^{-5}$	$4.3 \cdot 10^{-5}$	$5.2 \cdot 10^{-5}$
γ_1	2.03	1.86	1.89	1.96	1.94	1.90	2.16	1.94
γ_2	3.07	3.00	3.01	3.06	3.03	3.02	3.10	3.02
$-Q_0$	13.807	13.483	13.363	13.795	13.789	13.677	13.973	13.691
$-Q_1$	13.465	13.414	13.391	13.696	13.365	13.664	13.702	13.559
r	1.0254	1.0051	0.9979	1.0072	1.0317	1.0010	1.0198	1.0097
I_e	2.540	0.514	-0.209	0.723	3.172	0.095	1.978	0.974
**April**								
$P^D(45,1)$	$5.5 \cdot 10^{-5}$	$5.5 \cdot 10^{-5}$	$5.8 \cdot 10^{-5}$	$4.2 \cdot 10^{-5}$	$5.8 \cdot 10^{-5}$	$7.3 \cdot 10^{-5}$	$2.3 \cdot 10^{-5}$	$4.5 \cdot 10^{-5}$
γ_1	1.90	1.89	1.92	1.98	1.83	1.73	2.30	1.97
γ_2	2.99	3.00	3.01	3.06	2.93	2.89	3.23	3.02
$-Q_0$	13.622	13.622	13.542	13.919	13.586	13.370	14.497	13.828
$-Q_1$	13.201	13.547	13.557	13.811	13.177	13.425	14.075	13.703
r	1.0319	1.0055	0.9989	1.0078	1.0310	0.9959	1.0300	1.0091
I_e	3.189	0.554	-0.111	0.782	3.104	-0.410	2.998	0.912
**May**								
$P^D(45,1)$	$5.5 \cdot 10^{-5}$	$6.1 \cdot 10^{-5}$	$6.1 \cdot 10^{-5}$	$4.4 \cdot 10^{-5}$	$3.6 \cdot 10^{-5}$	$7.8 \cdot 10^{-5}$	$3.2 \cdot 10^{-5}$	$4.5 \cdot 10^{-5}$
γ_1	1.93	1.84	1.89	1.96	2.06	1.66	2.16	1.95
γ_2	2.98	2.94	2.98	3.04	3.04	2.85	3.16	3.02
$-Q_0$	13.633	13.522	13.505	13.864	14.069	13.270	14.197	13.815
$-Q_1$	13.652	13.593	13.568	13.931	14.088	13.360	14.298	13.841
r	0.9986	0.9948	0.9954	0.9952	0.9986	0.9933	0.9929	0.9981
I_e	-0.139	-0.522	-0.464	-0.481	-0.135	-0.674	-0.706	-0.188
**June**								
$P^D(45,1)$	$4.4 \cdot 10^{-5}$	$5.9 \cdot 10^{-5}$	$6.1 \cdot 10^{-5}$	$4.5 \cdot 10^{-5}$	$3.8 \cdot 10^{-5}$	$6.3 \cdot 10^{-5}$	$3.2 \cdot 10^{-5}$	$5.3 \cdot 10^{-5}$
γ_1	2.02	1.85	1.89	1.96	2.06	1.80	2.15	1.86

γ_2	3.05	2.96	2.96	3.00	3.01	2.90	3.14	2.96
$-Q_0$	13.890	13.568	13.511	13.855	14.058	13.543	14.229	13.671
$-Q_1$	14.180	13.608	13.656	13.943	14.162	13.671	14.423	13.738
r	0.9795	0.9971	0.9894	0.9937	0.9927	0.9906	0.9865	0.9951
I_e	-2.045	-0.294	-1.062	-0.631	-0.734	-0.9363	-1.345	-0.488

Table 3: Percentage of the total population of the provinces covered by the mortality data published by ISTAT, corresponding to the period 2015-2020.

**Bergamo**	**Brescia**	**Cremona**	**Lodi**	**Padova**	**Parma**	**Pesaro U.**	**Piacenza**
99.32%	99.75%	99.89%	99.07%	82.26%	98.86%	95.75%	99.96%

Table 4: Configurations "0" (average in 2015-2019) and "1" (2020) in the considered Italian provinces and Milano city. Weekly variation from February 2nd to June 13th 2020, derived from data released by ISTAT. Assumed probability of death $P^D(A_{r,2},\sigma) = 6.67 \cdot 10^{-6}$ at $A_{r,2} = 45$ solar years. Renormalization parameters γ_1 and γ_2 as *per* equation (2.1). Graphically-estimated values of $-Q_0$ and $-Q_1$ as *per* equation (2.2). Values of the *epidemic ratio* r and of the *relative index of epidemic* I_e.

	**Bergamo**	**Brescia**	**Cremona**	**Lodi**	**Milano**	**Padova**	**Parma**	**Pesaro**	**Piacenza**
**2-8/02**									
γ_1	2.20	2.21	2.30	2.30	2.26	2.20	2.19	2.08	2.20
γ_2	3.24	3.22	3.20	3.29	3.15	3.15	3.19	3.18	3.21
$-Q_0$	15.685	15.658	15.684	15.710	15.787	15.563	15.654	15.687	15.705
$-Q_1$	15.603	15.671	15.471	15.807	15.948	15.598	15.755	15.952	15.668
r	1.0053	0.9992	1.0138	0.9939	0.9899	0.9978	0.9936	0.9834	1.0024
I_e	0.526	-0.083	1.377	-0.614	-1.010	-0.224	-0.641	-1.661	0.236
**9-15/02**									
γ_1	2.26	2.20	2.16	2.15	2.22	2.20	2.25	2.20	2.25
γ_2	3.26	3.20	3.23	3.22	3.17	3.23	3.21	3.14	3.17
$-Q_0$	15.754	15.761	15.584	15.700	15.762	15.716	15.765	15.834	15.738
$-Q_1$	15.895	15.995	15.561	15.753	16.063	15.644	15.834	15.650	15.695
r	0.9911	0.9854	1.0015	0.9966	0.9813	1.0046	0.9956	1.0118	1.0027
I_e	-0.887	-1.463	0.148	-0.336	-1.874	0.460	-0.436	1.176	0.274
**16-22/02**									
γ_1	2.25	2.17	2.04	2.10	2.25	2.14	2.21	2.20	2.32
γ_2	3.25	3.20	3.24	3.20	3.15	3.23	3.15	3.21	3.18
$-Q_0$	15.783	15.725	15.660	15.636	15.831	15.677	15.804	15.724	15.847
$-Q_1$	15.939	15.794	15.470	15.595	15.931	15.818	16.020	15.914	16.035
r	0.9902	0.9956	1.0123	1.0026	0.9937	0.9911	0.9865	0.9881	0.9883
I_e	-0.979	-0.437	1.228	0.263	-0.628	-0.891	-1.348	-1.194	-1.172
**23-29/02**									
γ_1	2.20	2.10	2.22	2.17	2.17	2.12	2.16	2.09	1.96
γ_2	3.21	3.18	3.14	3.16	3.13	3.18	3.14	3.14	3.22
$-Q_0$	15.736	15.660	15.713	15.590	15.780	15.771	15.674	15.701	15.706
$-Q_1$	15.722	15.620	15.658	15.183	16.021	15.777	15.848	15.530	15.305
r	1.0009	1.0026	1.0035	1.0268	0.9850	0.9996	0.9890	1.0110	1.0262
I_e	0.089	0.256	0.351	2.681	-1.504	-0.038	-1.098	1.101	2.620
**1-7/03**									
γ_1	2.17	2.14	2.16	2.17	2.18	2.18	2.15	2.25	2.29
γ_2	3.20	3.22	3.22	3.20	3.10	3.23	3.17	3.15	3.15
$-Q_0$	15.608	15.681	15.687	15.691	15.681	15.741	15.577	15.685	15.799
$-Q_1$	14.843	15.493	15.005	14.651	15.825	15.980	15.328	15.811	15.380
r	1.0515	1.0121	1.0455	1.0710	0.9909	0.9850	1.0162	0.9920	1.0272
I_e	5.154	1.213	4.545	7.098	-0.910	-1.496	1.624	-0.797	2.724
**8-14/03**									
γ_1	2.20	2.10	2.12	2.22	2.14	2.15	2.20	2.15	2.18
γ_2	3.20	3.20	3.23	3.23	3.13	3.21	3.12	3.20	3.22
$-Q_0$	15.727	15.672	15.711	15.687	15.763	15.716	15.701	15.675	15.771
$-Q_1$	13.967	14.653	14.248	14.135	15.428	15.740	14.672	15.369	14.451
r	1.1260	1.0695	1.1027	1.1098	1.0217	0.9985	1.0701	1.0199	1.0913
I_e	12.601	6.954	10.268	10.980	2.171	-0.152	7.013	1.991	9.134
**15-21/03**									
γ_1	2.20	2.10	2.16	2.30	2.17	2.30	2.16	2.02	2.02

γ_2	3.22	3.20	3.22	3.22	3.10	3.15	3.16	3.18	3.20
$-Q_0$	15.702	15.641	15.633	15.826	15.749	15.816	15.693	15.637	15.625
$-Q_1$	13.520	14.234	13.960	13.958	15.260	15.580	14.282	14.913	14.401
r	1.1614	1.0988	1.1198	1.1338	1.0320	1.0151	1.0988	1.0485	1.0850
I_e	16.139	9.885	11.984	13.383	3.204	1.515	9.880	4.855	8.499
**22-28/03**									
γ_1	2.17	2.15	2.26	2.10	2.25	2.20	2.25	2.00	2.25
γ_2	3.20	3.17	3.17	3.20	3.10	3.17	3.14	3.19	3.12
$-Q_0$	15.688	15.780	15.851	15.744	15.879	15.677	15.698	15.734	15.733
$-Q_1$	13.538	14.102	13.995	14.143	15.160	15.721	14.494	14.666	14.352
r	1.1588	1.1190	1.1326	1.1132	1.0474	0.9972	1.0831	1.0728	1.0962
I_e	15.881	11.899	13.262	11.320	4.743	-0.280	8.307	7.282	9.622
**29-4/04**									
γ_1	2.16	2.15	2.18	2.40	2.22	2.14	2.18	2.08	2.10
γ_2	3.18	3.14	3.16	3.08	3.12	3.18	3.14	3.18	3.14
$-Q_0$	15.746	15.745	15.725	16.052	15.831	15.710	15.741	15.758	15.700
$-Q_1$	14.074	14.357	14.200	14.740	15.216	15.595	14.635	14.741	14.481
r	1.1188	1.0967	1.1074	1.0890	1.0404	1.0074	1.0756	1.0690	1.0842
I_e	11.880	9.668	10.739	8.901	4.042	0.737	7.557	6.899	8.418
**5-11/04**									
γ_1	2.22	2.19	2.23	2.22	2.14	2.17	2.07	1.97	2.23
γ_2	3.15	3.18	3.16	3.21	3.12	3.18	3.15	3.16	3.15
$-Q_0$	15.774	15.765	15.874	15.780	15.700	15.680	15.643	15.780	15.755
$-Q_1$	14.661	14.866	14.760	15.268	15.481	15.716	15.034	14.998	15.128
r	1.0759	1.0605	1.0755	1.0335	1.0141	0.9977	1.0405	1.0521	1.0414
I_e	7.591	6.047	7.547	3.353	1.415	-0.229	4.051	5.214	4.144
**12-18/04**									
γ_1	2.20	2.08	2.27	2.08	2.24	2.13	2.10	2.18	2.10
γ_2	3.15	3.15	3.13	3.22	3.09	3.18	3.12	3.12	3.15
$-Q_0$	15.743	15.830	15.801	15.857	15.674	15.785	15.679	15.638	15.951
$-Q_1$	15.080	15.044	15.024	14.960	15.420	15.654	15.125	15.214	14.980
r	1.0440	1.0522	1.0517	1.0600	1.0165	1.0084	1.0366	1.0279	1.0648
I_e	4.396	5.225	5.172	5.996	1.647	0.837	3.663	2.787	6.482
**19-25/04**									
γ_1	2.22	2.21	2.20	2.22	2.22	2.20	2.18	2.10	2.25
γ_2	3.15	3.11	3.15	3.16	3.06	3.14	3.11	3.14	3.11
$-Q_0$	15.737	15.801	15.895	15.754	15.670	15.695	15.609	15.534	15.870
$-Q_1$	15.348	15.370	15.204	15.607	15.600	15.916	15.446	15.465	15.555
r	1.0253	1.0280	1.0454	1.0094	1.0045	0.9861	1.0106	1.0045	1.0203
I_e	2.534	2.804	4.545	0.942	0.449	-1.388	1.055	0.446	2.025
**26-2/05**									
γ_1	2.15	2.12	2.12	2.10	2.12	2.10	2.08	1.95	2.06
γ_2	3.18	3.17	3.14	3.21	3.08	3.15	3.13	3.14	3.20
$-Q_0$	15.849	15.810	15.884	15.881	15.842	15.881	15.648	15.625	15.895
$-Q_1$	15.366	15.580	15.308	15.534	15.503	15.730	15.494	15.577	15.247
r	1.0314	1.0148	1.0376	1.0223	1.0219	1.0096	1.0099	1.0031	1.0425
I_e	3.143	1.476	3.763	2.234	2.187	0.960	0.994	0.308	4.250
**3-9/05**									
γ_1	2.20	2.12	2.00	2.12	2.18	2.25	2.13	2.10	2.23
γ_2	3.13	3.12	3.20	3.17	3.08	3.15	3.10	3.15	3.15
$-Q_0$	15.807	15.947	16.023	15.868	15.810	15.747	15.682	15.554	15.731
$-Q_1$	15.773	15.860	15.382	15.568	15.650	15.988	15.766	15.654	15.558
r	1.0022	1.0055	1.0417	1.0193	1.0102	0.9849	0.9947	0.9936	1.0111
I_e	0.215	0.549	4.167	1.927	1.022	-1.507	-0.533	-0.639	1.112

**10-16/05**									
γ_1	2.10	2.11	2.10	2.15	2.26	2.20	2.17	2.08	2.20
γ_2	3.19	3.15	3.13	3.09	3.05	3.16	3.10	3.16	3.14
$-Q_0$	15.774	15.857	15.874	15.837	15.690	15.740	15.783	15.631	15.871
$-Q_1$	15.710	15.780	15.431	15.827	16.033	16.002	15.864	15.440	15.718
r	1.0041	1.0049	1.0288	1.0006	0.9786	0.9836	0.9949	1.0124	1.0097
I_e	0.407	0.488	2.877	0.063	-2.139	-1.637	-0.511	1.237	0.973
**17-23/05**									
γ_1	2.12	2.17	2.20	2.17	2.12	2.17	2.18	2.16	2.28
γ_2	3.16	3.10	3.10	3.05	3.13	3.13	3.10	3.15	3.07
$-Q_0$	15.790	15.858	15.792	15.991	15.758	15.778	15.696	15.565	15.835
$-Q_1$	15.927	15.934	15.536	15.689	15.978	15.933	15.997	15.900	15.800
r	0.9914	0.9952	1.0165	1.0192	0.9862	0.9903	0.9812	0.9789	1.0022
I_e	-0.860	-0.477	1.648	1.925	-1.377	-0.973	-1.882	-2.107	0.222
**24-30/05**									
γ_1	2.14	2.15	2.21	2.22	2.22	2.09	2.10	2.18	2.14
γ_2	3.14	3.11	3.12	3.19	3.08	3.18	3.10	3.13	3.11
$-Q_0$	15.660	15.694	15.815	15.684	15.768	15.734	15.621	15.853	15.585
$-Q_1$	15.554	15.882	15.837	15.729	15.964	15.807	16.053	15.975	15.856
r	1.0068	0.9882	0.9986	0.9971	0.9877	0.9954	0.9731	0.9924	0.9829
I_e	0.681	-1.184	-0.139	-0.286	-1.228	-0.462	-2.691	-0.764	-1.709
**31-6/06**									
γ_1	2.15	2.20	2.16	2.17	2.18	2.18	2.15	2.22	2.08
γ_2	3.18	3.09	3.15	3.15	3.09	3.11	3.14	3.09	3.12
$-Q_0$	15.707	15.725	15.67	15.650	15.744	15.716	15.711	15.810	15.742
$-Q_1$	15.915	15.886	15.670	15.957	15.908	15.715	15.874	15.911	15.590
r	0.9869	0.9899	1.0000	0.9808	0.9897	1.0001	0.9897	0.9937	1.0097
I_e	-1.307	-1.013	0.000	-1.924	-1.031	0.006	-1.027	-0.635	0.975
**7-13/06**									
γ_1	2.20	2.17	2.15	2.10	2.20	2.15	2.23	2.15	2.18
γ_2	3.15	3.11	3.13	3.13	3.09	3.15	3.05	3.05	3.11
$-Q_0$	15.752	15.731	15.580	15.582	15.845	15.790	15.702	15.979	15.612
$-Q_1$	15.696	15.889	16.043	15.921	15.940	15.726	16.048	15.854	15.647
r	1.0036	0.9901	0.9711	0.9787	0.9940	1.0041	0.9784	0.9964	0.9978
I_e	0.357	-0.994	-2.886	-2.129	-0.596	0.407	-2.156	-0.360	-0.224

Table 5: Values of the *relative index of epidemic* I_e in March 2020 and comparison with the indicator $D-1 = N_{d,20}/N_{d,15-19} - 1$, where $N_{d,20}$ and $N_{d,15-19}$ are, respectively, the number of deaths in March 2020 and the mean number of deaths in the same month of the years 2015-2019. Mortality data released by ISTAT.

	**Abruzzo**	**Campania**	**Emilia R.**	**Friuli V.G.**	**Lazio**	**Liguria**	**Lombardia**	**Marche**
I_e	1.097	-2.315	3.778	0.537	-0.292	2.046	7.167	2.573
$D-1$	0.160	0.024	0.717	0.158	0.025	0.557	1.913	0.493
	**Piemonte**	**Puglia**	**Sicilia**	**Toscana**	**Trentino A.A.**	**Umbria**	**Valle d'Aosta**	**Veneto**
I_e	2.540	0.514	-0.209	0.723	3.172	0.095	1.978	0.974
$D-1$	0.543	0.122	0.025	0.141	0.698	0.094	0.609	0.252
